# Layered Nickel–HHTP Networks for Efficient Chemiresistive Sensing of CH_4_ and CO_2_

**DOI:** 10.1002/smll.202600029

**Published:** 2026-08-22

**Authors:** Ignasi Fort‐Grandas, Marta Martínez‐Belmonte, Jordi Benet‐Buchholz, Rosa M. Gomila, Antonio Frontera, Albert Romano‐Rodríguez, Anton Vidal‐Ferran

**Affiliations:** ^1^ Department of Electronic and Biomedical Engineering Universitat De Barcelona Barcelona Spain; ^2^ Institute of Nanoscience and Nanotechnology (IN^2^UB) Universitat de Barcelona Barcelona Spain; ^3^ Department of Inorganic and Organic Chemistry Universitat De Barcelona Barcelona Spain; ^4^ Institute of Chemical Research of Catalonia (ICIQ) Tarragona Spain; ^5^ Department of Chemistry Universitat De Les Illes Balears Palma de Mallorca Spain; ^6^ ICREA (Institució Catalana de Recerca i Estudis Avançats) Barcelona Spain

**Keywords:** coordination chemistry, chemiresistive greenhouse gas sensing, HHTP‐based coordination compounds, MOF, supramolecular solid‐state chemistry

## Abstract

The synthesis and single‐crystal structural elucidation of Ni‐HHTP are reported for the first time, achieved from crystals of sufficient size for electron diffraction analysis. These studies reveal that the crystal structure of Ni‐HHTP alternates between radical‐rich and closed‐shell layers along the *c*‐axis, forming a periodic, sandwich‐like architecture. The Ni‐HHTP coordination pattern differs between these two types of alternating 2D‐networks, reflecting distinct electronic environments. Water molecules are either coordinated to the framework or accommodated within the lattice, giving rise to a swelling effect with increasing water content. The well‐defined structure enables direct correlation between framework architecture and function. Ni‐HHTP exhibits a distinct chemiresistive response toward CO_2_ and CH_4_—gases of high relevance to greenhouse gas monitoring. Notably, the sensor delivers a clear and measurable response to both gases at concentrations below their ambient atmospheric levels. Theoretical calculations reveal the nature of intramolecular interactions governing the sensing process, providing molecular‐level insight into the chemiresistive behavior of Ni‐HHTP‐based gas sensors.

## Graphical abstract

First single‐crystal structure of Ni‐HHTP reveals alternating radical‐rich and closed‐shell layers forming a periodic, sandwich‐like framework. Coordinated and lattice water modulate interlayer spacing. The defined structure enables correlation between architecture and chemiresistive sensing toward CO_2_ and CH_4_, supported by theoretical insight into molecular interactions.

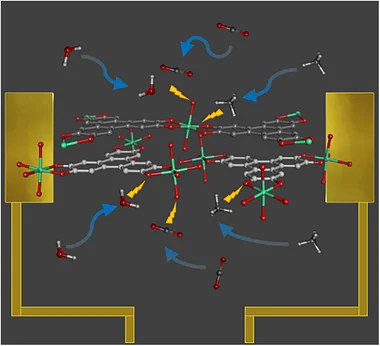

## 1 Introduction

Among the many materials discovered in recent years, metal‐organic frameworks (MOFs) have emerged as particularly noteworthy. Composed of metal ions or clusters linked by organic ligands, MOFs possess large surface areas and permanent porosity, making them excellent platforms for molecular adsorption, separation, and catalytic applications [1, 2, 3, 4, 5, 6]. The properties of MOFs can be tuned through control of the type, ratio, and spatial arrangement of metal ions and organic ligands. This strategy has enabled the development of MOFs with enhanced functionalities, such as improved electrical conductivity, thereby opening new avenues for electronic applications [7, 8, 9].

Several conductive MOFs featuring structurally diverse ligands have been reported [7, 9, 10]. Of particular relevance to the present work are MOFs constructed from triphenylene‐2,3,6,7,10,11‐hexaol (HHTP), a widely used precursor in the synthesis of conductive frameworks. HHTP consists of three benzene‐1,2‐diol moieties symmetrically arranged around a central benzene ring, giving rise to a rigid, planar, and highly conjugated architecture. The coordination chemistry of the benzene‐1,2‐diol moiety with divalent metal ions is well documented in the literature [11]. In the absence of a strong base [12] and/or in the presence of an oxidizing agent, benzene‐1,2‐diol reacts with divalent metal precursors including Ni(II) derivatives to form homoleptic bis‐*ortho*‐semiquinonate metal(II) complexes [13]. During the coordination process, each benzene‐1,2‐diol unit is oxidized to its semiquinonate form—a monoanionic, monoradical species—which serves as the actual coordinating ligand to the metal center. Interestingly, the resulting complexes are electrically neutral and possess two unpaired electrons, with one localized on each benzene‐1,2‐diol unit. Applying this type of coordination chemistry to HHTP—which, as previously noted, features three benzene‐1,2‐diol units—enables the construction of infinite two‐dimensional MOFs presenting hexagonal cavities. The first synthesis of such 2D MOFs, Co‐HHTP [14] and Ni‐HHTP, was reported by Yaghi et al. in 2012 [15]. These materials were prepared by combining HHTP with cobalt(II) or nickel(II) metal precursors in a 2:3 molar ratio. This pioneering study, along with subsequent research on this type of 2D‐MOFs incorporating other metals (for the synthesis and properties of Cu‐HTTP, see references [16] and [17]), revealed that the benzene‐1,2‐diol coordination sites promote efficient electronic communication across the triphenylene core due to the presence of unpaired electrons and extended conjugation [18]. Additionally, the formation of strong metal‐ligand bonds facilitates charge delocalization between the ligand and metal centers, collectively endowing these MOFs with intrinsic electrical conductivity [7]. Besides the in‐plane conductivity in triphenylene‐derived MOFs, some studies suggest that the through‐plane pathway can also play a significant role in their electric conductivity [17].

Up to date, few crystallographic studies have been reported in the literature from triphenylene‐derived MOFs [15, 19]. These studies reveal that, despite utilizing the same HHTP ligand, the resulting MOF structures vary significantly depending on the metal center employed. Notably, the single‐crystal structure of Ni‐HHTP has not yet been reported [20], despite its excellent electrical conductivity and its status as one of the most widely studied triphenylene‐based MOFs [21].

Triphenylene‐derived MOFs have attracted considerable attention for a variety of electronic applications, owing to their intrinsic electrical conductivity. They have been investigated as electrode materials for supercapacitors and batteries [8, 22], as well as for their potential in electrocatalysis [8, 22] and chemiresistive sensing [10, 23, 24, 25] of analytes including gases. However, many sensors of this type still suffer from slow recovery, high detection limits, or poor analyte selectivity, limiting their practical utility. In this work, we report the controlled synthesis and single‐crystal structural characterization of Ni‐HHTP, with its structure determined by electron diffraction. We also investigate the chemiresistive sensing properties of Ni‐HHTP toward two major greenhouse gases, namely CO_2_ and CH_4_. Furthermore, we present theoretical calculations that shed light on the sensing mechanism, elucidating the nature of the intramolecular interactions during the sensing event. These insights are critical for advancing the understanding of the chemiresistive behavior of Ni‐HHTP‐based gas sensors.

## 2 Results and Discussion

### 2.1 Ni‐HHTP Synthesis and Crystal Structure Determination

HHTP‐based MOFs have previously been synthesized using diverse strategies, often resulting in polycrystalline solids with inconsistent crystallinity [4]. Among the available approaches, solvothermal synthesis has proven to be the most effective route for obtaining crystalline materials [15]. Nevertheless, the outcome of this process is highly sensitive to numerous experimental variables, where factors such as sonication of precursor solutions, the use of co‐solvents with water, and the order of reagent addition critically influence crystallinity. Owing to this inherent complexity, no synthetic protocol can be anticipated a priori to yield crystalline material. In this work, we established and optimized a solvothermal strategy for Ni‐HHTP, integrating controlled sonication steps and the use of DMF as a coordinating solvent to promote crystal growth and structural order. In brief, the HHTP ligand (limiting reagent) was added at once to an aqueous solution of nickel acetate (1.15 equiv.) and sonicated (10 min.). *N*,*N*‐dimethylformamide (DMF), a coordinating solvent, was then added dropwise (0.1 mL/min). After an additional sonication step (10 min.), the mixture was heated at 80°C for 6 h. Crystals were isolated by centrifugation, washed with water and ethanol, and vacuum‐dried to afford the final product (see Supporting Information for details). Direct microscopic observation revealed a crystalline material composed of numerous small monocrystals (see Figure 1a and Figure SI1). Although these crystals were too small for conventional single‐crystal X‐ray diffraction analysis, their size and properties were well‐suited for electron diffraction studies. This technique enabled the unequivocal determination of the material's structure (see Tables SI1–SI5 and Table SI9).

**FIGURE 1:**
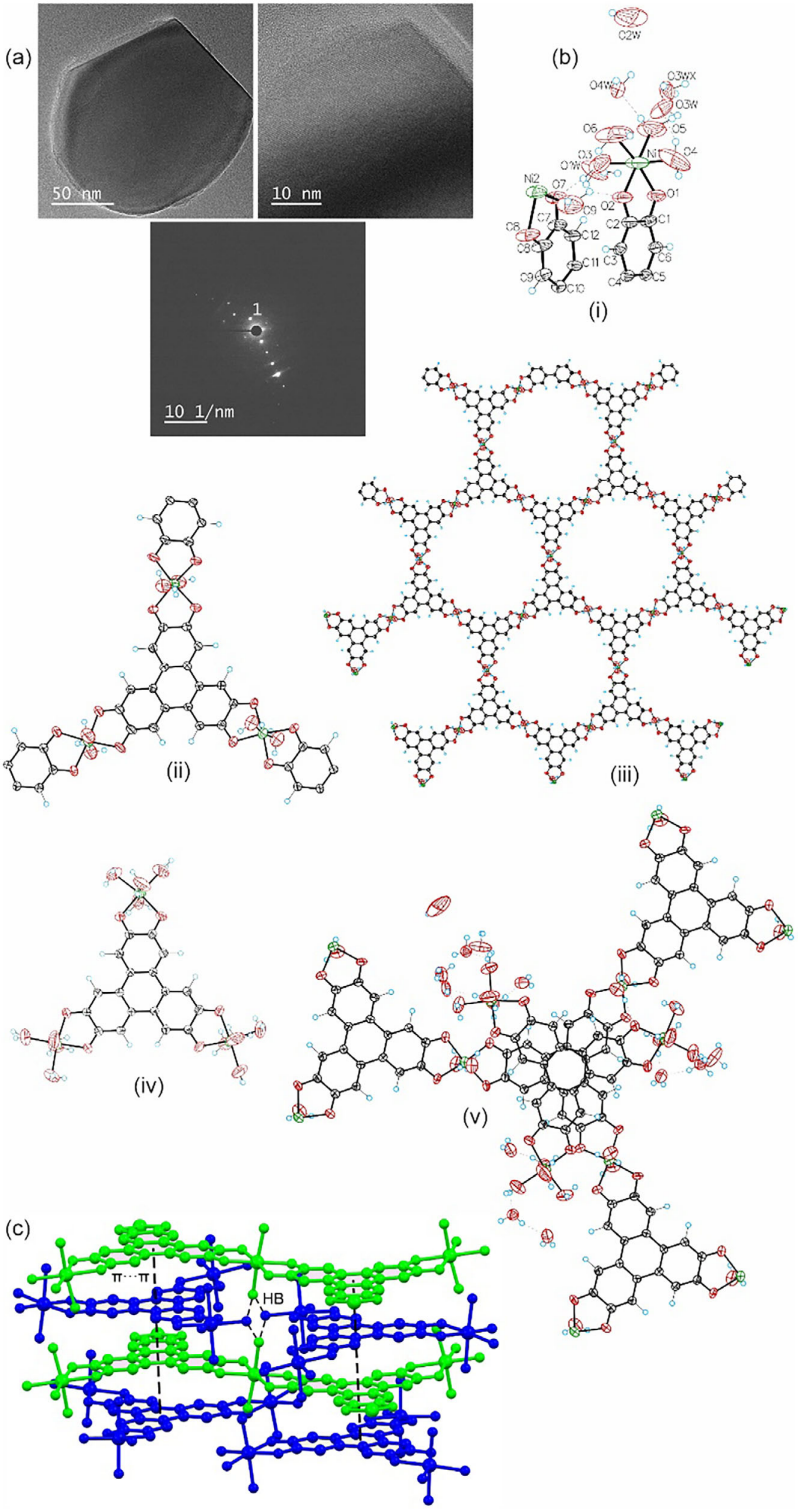
(a) Ni‐HHTP TEM images of the MOF single crystal, with the electron diffraction pattern: interplanar distance retrieved from diffraction spot “1“ is 3.157 Å. (b) ORTEP drawings (thermal ellipsoids at 50% probability) showing: (i) asymmetric unit with formula C_12_H_16.8_Ni_1.5_O_10.4_ (non‐integer atom counts arise from partially occupied atomic sites within the asymmetric unit); (ii) a structural fragment featuring nickel centers in the first coordination mode (Ni_3_(HHTP)_2_·6H_2_O‐based network); (iii) their extended coordination network; (iv) a structural fragment featuring nickel centers in the second coordination mode (Ni_3_(HHTP)_1_·12H_2_O‐based network) and (v) Ni_3_(HHTP)_1_·12H_2_O fragment superimposed to an extended Ni_3_(HHTP)_2_·6H_2_O‐based network. (c) Schematic representation of the two alternating Ni‐HHTP layers in the crystal structure. Green and blue highlight layers based on the first and second coordination modes of HHTP with Ni, respectively.

The crystalline material was identified as belonging to the trigonal crystal system, specifically the space group P‒3c1, with lattice parameters *a* = *b* = 21.62(14) Å and *c* = 12.70(19) Å. The crystal features a relatively large unit cell, with a volume of 5137(101) Å^3^ containing approximately 500 atoms. A key structural feature is the coexistence of two distinct coordination modes between nickel centers and the HHTP ligand, each giving rise to different local geometries and charge states. In the first coordination mode, nickel ions are coordinated by two in‐plane HHTP molecules. This coordination mode involves partial deprotonation of HHTP to yield a trianionic monoradical ligand (for the synthesis and properties of Cu‐HTTP, see references [16, 17] and [26] and for the electrochemical characterization of triphenylene‐2,3,6,7,10,11‐hexaol, see references [27, 28]). Two water molecules coordinated to each nickel center complete the octahedral environment, giving rise to the Ni_3_(HHTP)_2_·6H_2_O core (for the basic coordination motif (ii) and the extended network (iii), see Figure 1b). The resulting geometry confirms the presence of out‐of‐plane water molecules. This coordination arrangement generates large hexagonal cavities, which give rise to the honeycomb‐like architecture. In the second coordination mode, all three benzene‐1,2‐diol moieties of HHTP are fully deprotonated, forming a non‐radical hexaanionic ligand that coordinates to three nickel centers. Each nickel in this second coordination mode exhibits an octahedral environment, with one benzene‐1,2‐diolate unit occupying two coordination sites and four water molecules completing the octahedral geometry. This coordination arrangement results in the formation of a Ni_3_(HHTP)_1_·12H_2_O core (for the basic coordination motif (iv), and its relative spatial arrangement with the Ni_3_(HHTP)_2_·6H_2_O‐based network (v), see Figure 1b). The number of Ni_3_(HHTP)_2_·6H_2_O cores in the crystal is equal to that of Ni_3_(HHTP)_1_·12H_2_O. For a view of the whole crystalline structure, see Figure SI16.

As discussed earlier, Figure 1b‐iii displays the extended structure of the Ni_3_(HHTP)_2_·6H_2_O‐based framework. In this structure, coordination of the nickel centers by two semiquinonate units from the HHTP ligand, each containing an unpaired electron, gives rise to a Kagome lattice [29]. This structural motif is commonly associated with triphenylene‐based MOFs. This extended, conjugated, and polyradical two‐dimensional layer derived from Ni_3_(HHTP)_2_·6H_2_O (see Figure 1b‐iii and green layer in Figure 1c) associates above and below with discrete, closed‐shell Ni_3_(HHTP)_1_·12H_2_O units through *π–π* [30] stacking and hydrogen‐bonding interactions [31]. This arrangement results in the formation, along the *c* axis, of two unconjugated two‐dimensional networks derived from Ni_3_(HHTP)_1_·12H_2_O units (blue layers in Figure 1c), one positioned below and the other above the radical‐rich HHTP‐based layer. The overall structure thus adopts a periodic, sandwich‐like architecture. HHTP motifs associated with both nickel coordination environments are positioned either within or in proximity to the (004) crystallographic planes. The separation between two adjacent Ni_3_(HHTP)_1_·12H_2_O‐based or Ni_3_(HHTP)_2_·6H_2_O‐based networks are approximately 6.3 Å. On the other hand, the separation between HHTP motifs in consecutive layers varies from ca. 3.0 to 3.3 Å (for the distances between HHTP units within the crystal structure, see Figure SI17).

The present work provides the first comprehensive crystallographic characterization of Ni‐HHTP. Yaghi [15] proposed, based on powder X‐ray diffraction and electron microscopy, that Ni‐HHTP is isostructural with Co‐HHTP, whose structure was determined by single‐crystal X‐ray diffraction. However, no direct single‐crystal structure determination of Ni‐HHTP was reported. Our results confirm that Ni‐HHTP and Co‐HHTP are isostructural, exhibiting closely similar unit‐cell parameters and volumes [20]. In the present study, all water molecules, including both Ni‐coordinated and pore‐disordered molecules, were refined anisotropically and all hydrogen atoms were located. In contrast, water molecules in the Co‐HHTP analogue were refined isotropically with their hydrogen atoms not being located [15]. The present study therefore provides the first complete structural description of Ni‐HHTP, including all relevant atomic connectivities and coordination features. Moreover, the geometric parameters within the Ni‐HHTP layers and the unit‐cell dimensions (discussed in the next section) are strongly dependent on water content. Single crystals of Ni‐HHTP were characterized by transmission electron microscopy (TEM), as shown in Figure 1a. Measurement of the interplanar spacing by TEM (3.157 Å) closely matches the 3.190 Å spacing associated with the (004) crystallographic planes, which accommodate HHTP motifs from both nickel coordination modes.

### 2.2 Impact of Humidity on the MOF Structure

The crystal structure of Ni‐HHTP revealed a high content of water molecules, with some incorporated into the framework and others occupying the pores and interlayer spaces. Because these water molecules may influence the structural stability and gas‐sensing behavior of the material, we investigated structural changes as a function of relative humidity (RH) at room temperature. The resulting X‐ray diffraction patterns (Figure 2a) show a systematic shift of all peaks toward lower angles with increasing RH, indicating lattice expansion, i.e., swelling upon water uptake. A closer examination of the diffraction profiles around 2θ ≈ 9.3° (Figure 2b), corresponding to the (200) reflections, and 2θ ≈ 27° (Figure 2c), corresponding to the (004) and (104) reflections, clearly highlights this behavior. The plots of the (200) and (004) interplanar distances as a function of relative humidity (Figure 2d), together with the evolution of the lattice parameters *a* and *c* (Figure 2e) — or their variation expressed as a percentage relative to the value at 10% RH (Figure 2f) — clearly indicate the swelling of the crystal upon water uptake.

**FIGURE 2:**
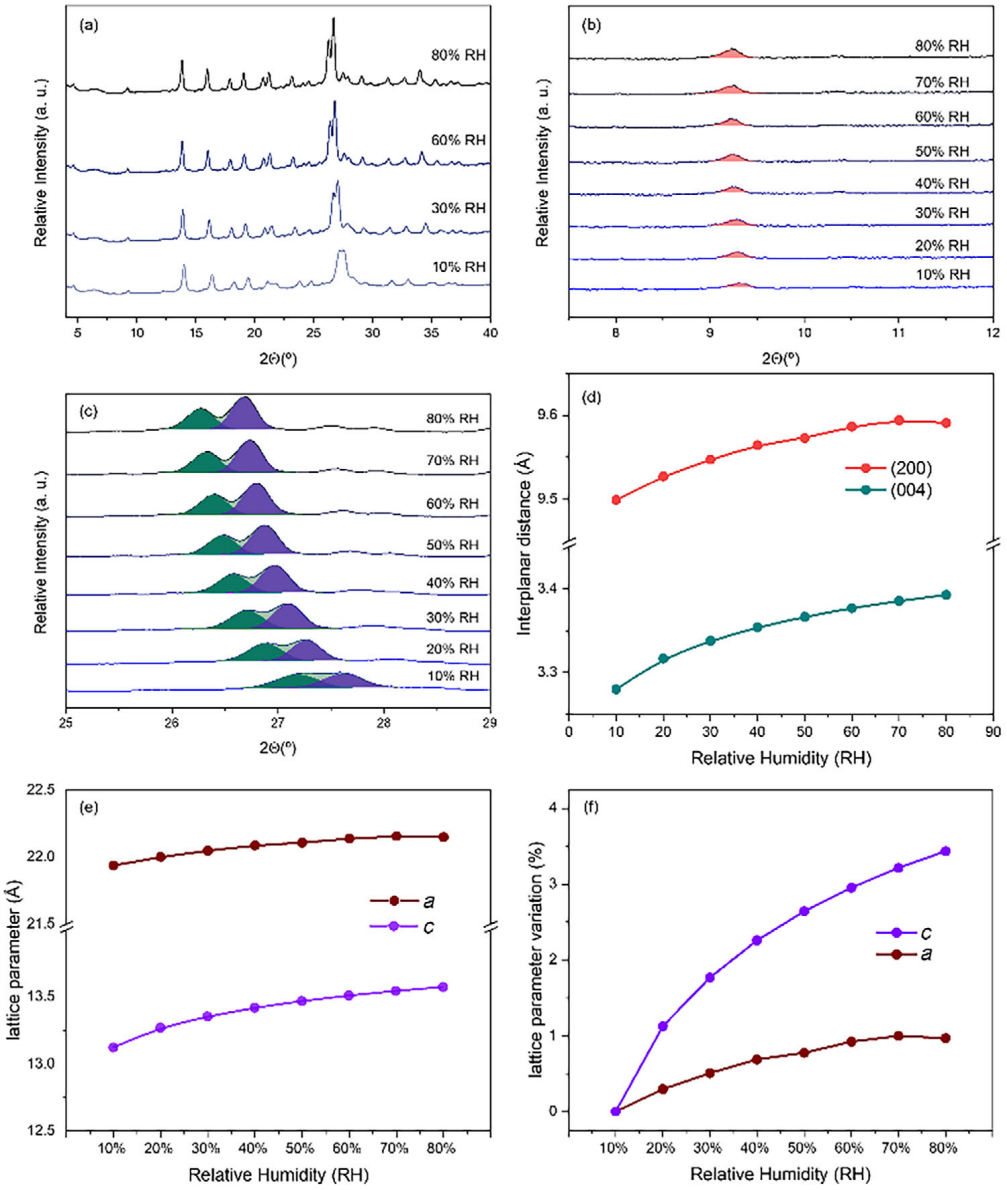
(a) PXRD patterns of Ni‐HHTP as a function of the RH at 25°C. In red, PXRD patterns of Ni‐HHTP at different RH with deconvoluted fitting of the (200) peaks (b) and, in dark cyan and red, respectively, the deconvoluted fitting of the (004)/(104) peaks (c). (d) (200) and (004) interplanar retrieved distances as a function of RH. (e) Crystal lattice parameters, *a* and *c*, deduced from (b). (f) Lattice parameter variation expressed as a percentage relative to the value at 10% RH. Solid lines correspond to eye guidelines.

This behavior highlights the material's pronounced sensitivity to initial water uptake, likely arising from strong interactions between water molecules and the framework under unsaturated conditions. In contrast, at higher RH values, the incremental structural change slows down, consistent with the approach to a saturation regime in which additional water adsorption has only a negligible influence on the lattice dimensions. Notably, this expansion is proportionally larger along the *c* axis (Figure 2f), and its implications for the electrical properties of the material will be discussed in the following section. Interestingly, the incorporation and release of water molecules is fully reversible, as evidenced by the recovery of the diffraction patterns to their original state upon cycling the humidity (see Figure SI4).

### 2.3 Computational Insights into the Interactions of Ni‐HHTP With H_2_O, CO_2_ and CH_4_

The ability of Ni‐HHTP to interact with small guest molecules such as H_2_O, CO_2_ and CH_4_ was probed computationally. A simplified model was developed to rationalize the structural complexity of the system, which comprises two alternating layers: an extended, conjugated two‐dimensional framework with radical semiquinonate coordination, and a discrete layer of trianionic units assembled into an extended network through noncovalent interactions. The model used for our computational studies consisted of two subunits. The first subunit (Fragment A, Figure 3a) includes one HHTP ligand and three Ni(II) centers, each coordinated by a semiquinonate anion radical, representing a fragment of the Ni‐HHTP framework based on Ni_3_(HHTP)_2_·6H_2_O. The second subunit (Fragment B, Figure 3b) corresponds to a discrete Ni_3_(HHTP)_1_·12H_2_O unit featuring a hexaanionic ligand and three Ni(II) centers, each completing an octahedral geometry through coordination with four water molecules [32].

**FIGURE 3:**
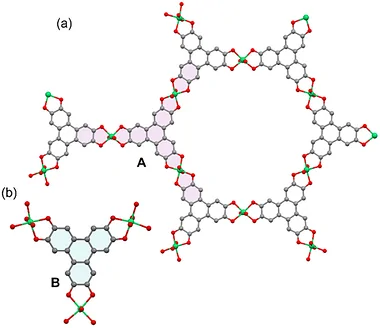
(a) Partial view of the infinite polymeric MOF layer based on Ni_3_(HHTP)_2_·6H_2_O, with the modelled subunit highlighted in fuchsia (Fragment A). (b) Discrete units of the second MOF layer, based on Ni_3_(HHTP)_1_·12H_2_O, with the modelled subunit highlighted in light blue (Fragment B).

To understand Ni‐HHTP's selectivity for different guests, the Molecular Electrostatic Potential (MEP) of the MOF fragments and guest molecules (N_2_, CH_4_, CO_2_ and H_2_O) was computed. N_2_ was included in the calculations to mimic the synthetic‐air atmosphere used in the sensing experiments, while H_2_O was computed to account for the substantial water content present within the pores and interlayer spaces. The MEP analysis for Fragment A (Figure 4a) reveals its maximum positive potential at the hydrogen atoms of the Ni‐coordinated water molecules (+43.9 kcal/mol) and its minimum at the Ni‐coordinated oxygen atoms of the organic ligand (–32.0 kcal/mol). The MEP over the aromatic ligand is weak, suggesting a propensity for π‐stacking without significant electrostatic repulsion. In sharp contrast, Fragment B (Figure 4b) shows its minimum sharply localized over the central aromatic ring (–50.8 kcal/mol), unlike the electroneutral character of the same region in Fragment A. Another key difference is that the MEP at the aromatic H‐atoms is negative in Fragment B (–17.6 kcal/mol) instead of positive as in Fragment A (+15.1 kcal/mol). Critically, in the solid state, the aromatic rings of Fragments A and B are stacked, making these surfaces unavailable for interactions with guest molecules and thus not relevant for rationalizing the Ni‐HHTP's adsorption properties. The MEP surfaces of the small guest molecules are also displayed in Figure 4. N_2_ exhibits moderate potentials (–9.8 and +8.2 kcal/mol), while CH_4_ shows weak, overall positive character. CO_2_ exhibits a pronounced electrophilic character at the carbon atom (+24.2 kcal/mol), and H_2_O displays strong, complementary potentials, with minima at the lone pairs (–35.6 kcal/mol) and maxima at the hydrogens (+41.9 kcal/mol), consistent with its known hydrogen‐bonding capability [31].

**FIGURE 4:**
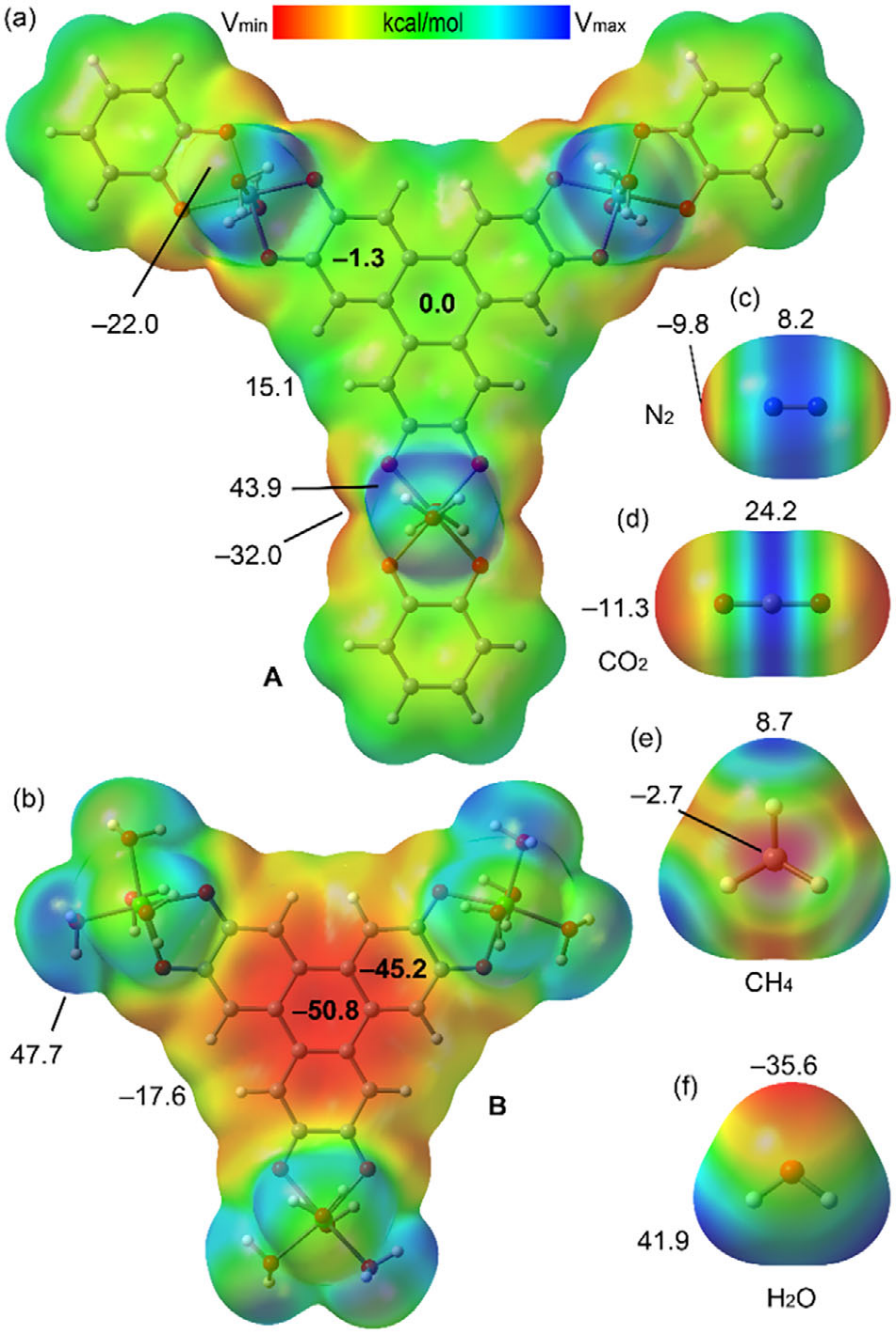
MEP surfaces of Fragment A (a), Fragment B (b), N_2_ (c), CO_2_ (d), CH_4_ (e), and H_2_O (f). Values at selected points are given in kcal/mol. Isovalue 0.001 a.u.

The MEP analysis suggests the strongest interactions would occur between the coordinated water molecules in Ni‐HHTP and guest H_2_O. However, the 3D architecture reveals that both the aromatic surfaces and the coordinated water molecules are involved in crystal packing, rendering them unavailable for guest interactions, as shown in Figure 1c. This limited accessibility must be considered when interpreting the subsequent results.

The interaction energies of small molecules with Fragment A were computed to determine binding preferences. The results, summarized in Figure 5, show that N_2_ interacts very weakly (–0.3 kcal/mol), confirming that its use in air as a carrier gas does not produce a competitive binding effect. Methane interacts primarily with the accessible oxygen atoms of the aromatic ligand coordinated to Ni(II). In contrast, the full optimization of Fragment A with CO_2_ leads to a global minimum where the CO_2_ interacts with both the coordinated water molecules and the ligand's oxygen atoms. This results in a relatively high interaction energy (–7.0 kcal/mol, see Figure SI18a) due to a combination of hydrogen bonds and O···C contacts. However, this global minimum involves coordination sites that are sterically hindered or occupied by the framework connectivity in the solid state. Since the coordinated water molecules are unavailable in the actual Ni‐HHTP structure, Figure 5c includes the optimized geometry and interaction energy of a local minimum where CO_2_ interacts with the aromatic ligand in a conformation similar to N_2_. In this case, the interaction energy is significantly lower (–0.9 kcal/mol). This lower value for the structurally accessible site explains the modest experimental adsorption of CO_2_ compared to CH_4_ and aligns the theoretical model with the observed sensor response. Water exhibits the strongest binding energy (–17.5 kcal/mol) due to the formation of three strong hydrogen bonds—acting once as an acceptor with a Ni‐coordinated water molecule and twice as an acceptor with the Ni‐coordinated oxygen atoms of the organic ligand. This observation accounts for the hydrated state of the MOF after synthesis and strongly indicates that the lattice water molecules remain within the structure during the experiments, without being displaced by CH_4_ or CO_2_.

**FIGURE 5:**
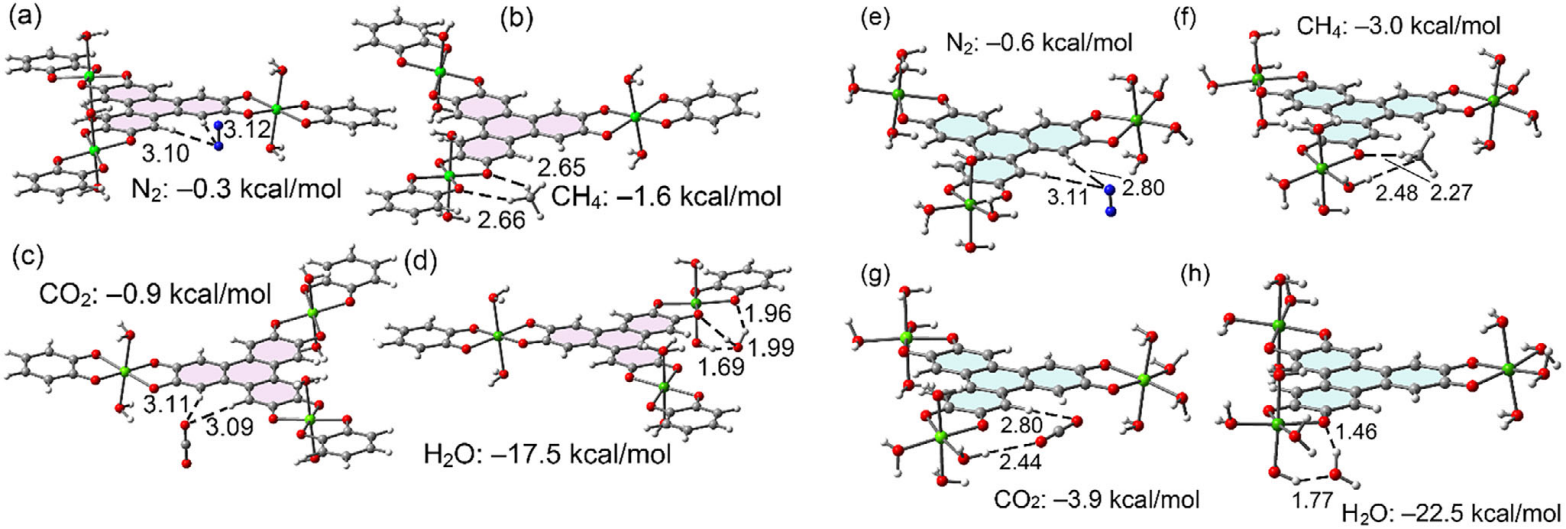
Complexes of Fragment A with N_2_ (a), CH_4_ (b), CO_2_ (c), and H_2_O (d) and those of Fragment B with N_2_ (e), CH_4_ (f), CO_2_ (g), and H_2_O (h). Distances are indicated in Å and binding energies in kcal/mol.

The same analysis was carried out for Fragment B in interaction with the small molecules. The results presented in Figure 5 reveal that this fragment interacts more strongly with all guest molecules than Fragment A. A key geometric difference is observed for CH_4_, which forms only one CH···O contact due to the different environment of the Ni metal centers. However, the interaction energy is larger with Fragment B because of an additional OH···C contact between a water proton and the carbon atom of methane, which has a negative MEP value. A second key geometric difference is found in the local minimum for CO_2_ (see Figure SI18b for the global minimum), where the CO_2_ molecule is coplanar, forming two H‐bonds with an aromatic CH group and one equatorial water molecule (Figure 5g). The binding energy is slightly more negative than that of CH_4_ (–3.9 kcal/mol). Nevertheless, considering the interactions of CH_4_ and CO_2_ with both fragments A and B collectively, the theoretical results support a higher affinity for CH_4_ over CO_2_. This conclusion is based not only on the binding energies of the structurally accessible local minima but also on the architectural dominance of Fragment A. As Fragment A constitutes the continuous, extended framework and defines the large hexagonal pores of the MOF, its specific binding preference for CH_4_ carries more weight in determining the overall sensor response than the discrete units of Fragment B.

### 2.4 Determination of Bandgap and Electrical Properties of the Pristine Ni‐HHTP

The light absorption of Ni‐HHTP has been studied through UV–vis absorption spectroscopy. As shown in Figure 6a, the material presents a strong absorption for wavelengths below 450 nm. Using these data, it is possible to determine the bandgap using the Tauc plot method [33]. The best fitting, shown in Figure 6b, is obtained assuming that Ni‐HHTP possesses a direct bandgap, with a value of Eg = 2.90 eV. Both the value and the bandgap type are in agreement with data from literature [34]. It is worth noting that in the previous reference [34], the MOF material was in the form of a continuous film, whereas in the present study, Ni‐HHTP was used as a powder.

**FIGURE 6:**
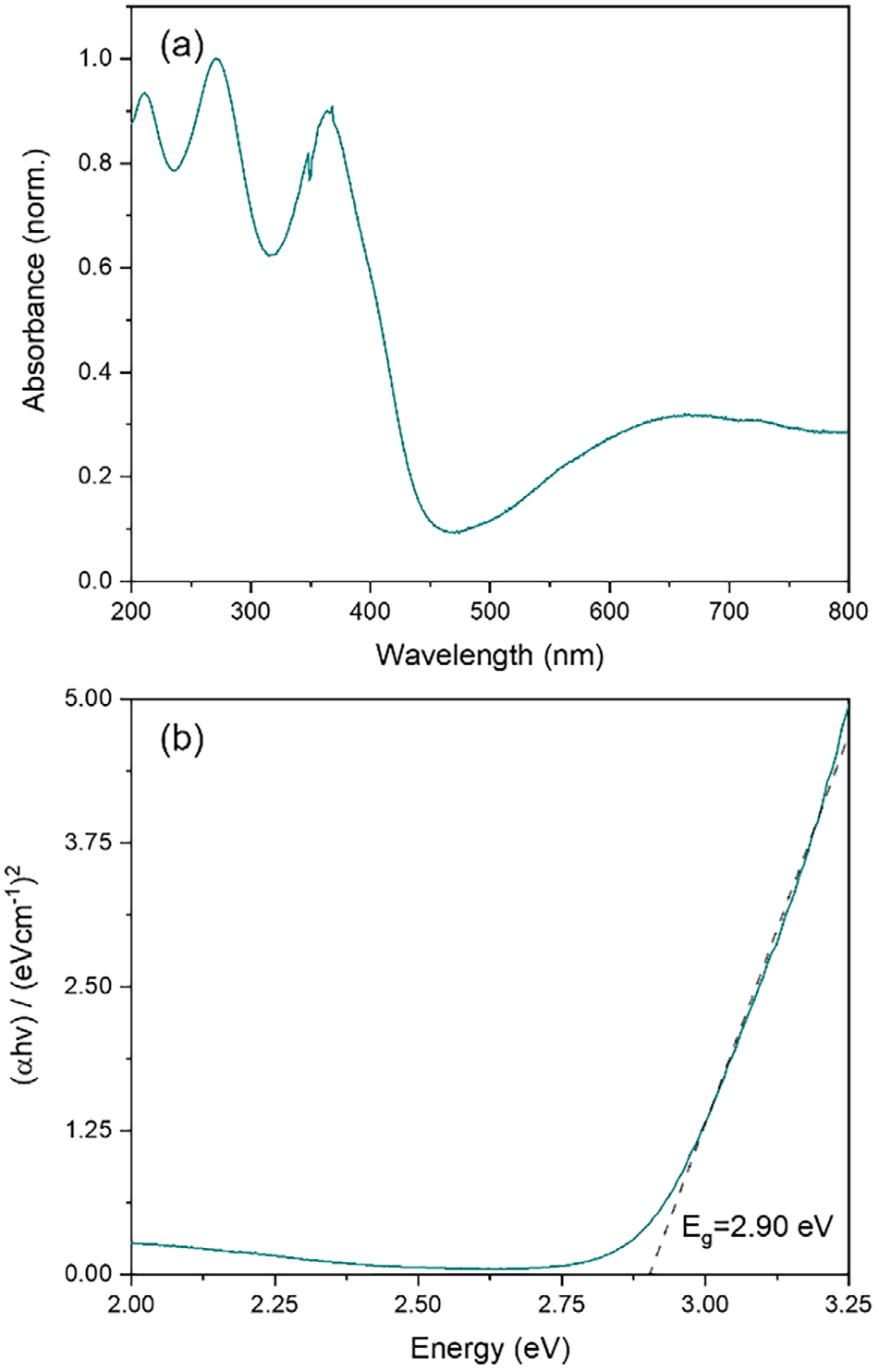
(a) UV–vis absorption spectrum of the Ni‐HHTP powder. (b) The corresponding Tauc plot, calculated assuming a direct bandgap semiconductor. The obtained value is 2.9 eV. Solid lines correspond to eye guidelines.

To carry out the electrical characterization of the material, two drops of a solution of Ni‐HHTP (2 mg/mL in deionized water) were drop casted on top of chips containing Cr/Au interdigitated electrodes (IDE) (for the description of the chip fabrication process and its layout, see the Supporting Information and Figures SI5 and SI6 there). The deposition of Ni‐HHTP onto the IDE forms an electrical bridge between the gold electrodes, enabling the characterization of the Au/Ni‐HHTP interface (Figure SI5). The room‐temperature (23°C) current‐voltage (I‐V) curves recorded at different relative humidity values are shown in Figure 7a. This figure clearly shows a perfectly linear relation between current and voltage, which confirms that the contacts between the Au electrodes and the MOF are ohmic. This observation—namely, the absence of rectifying (Schottky) behavior is consistent with literature reports for single crystals [35] and enables the electrical response of the material in the presence of gases to be interpreted solely in terms of gas‐MOF interactions. The measurements started at an ambient relative humidity (RH) of 33%. Next, the RH was lowered to 4% and, from this value, it was increased monotonously up to 78% and, finally, the initial 4% RH was recovered. The obtained resistance for each RH value is plotted in Figure 7b. From these results, it can be concluded that, on the one hand, the resistance increases with atmospheric water and, on the other, this effect is reversible within the conditions explored in this experiment. This reversibility is a positive indication for the use of this material in gas sensing applications, as will be shown in the following section.

**FIGURE 7:**
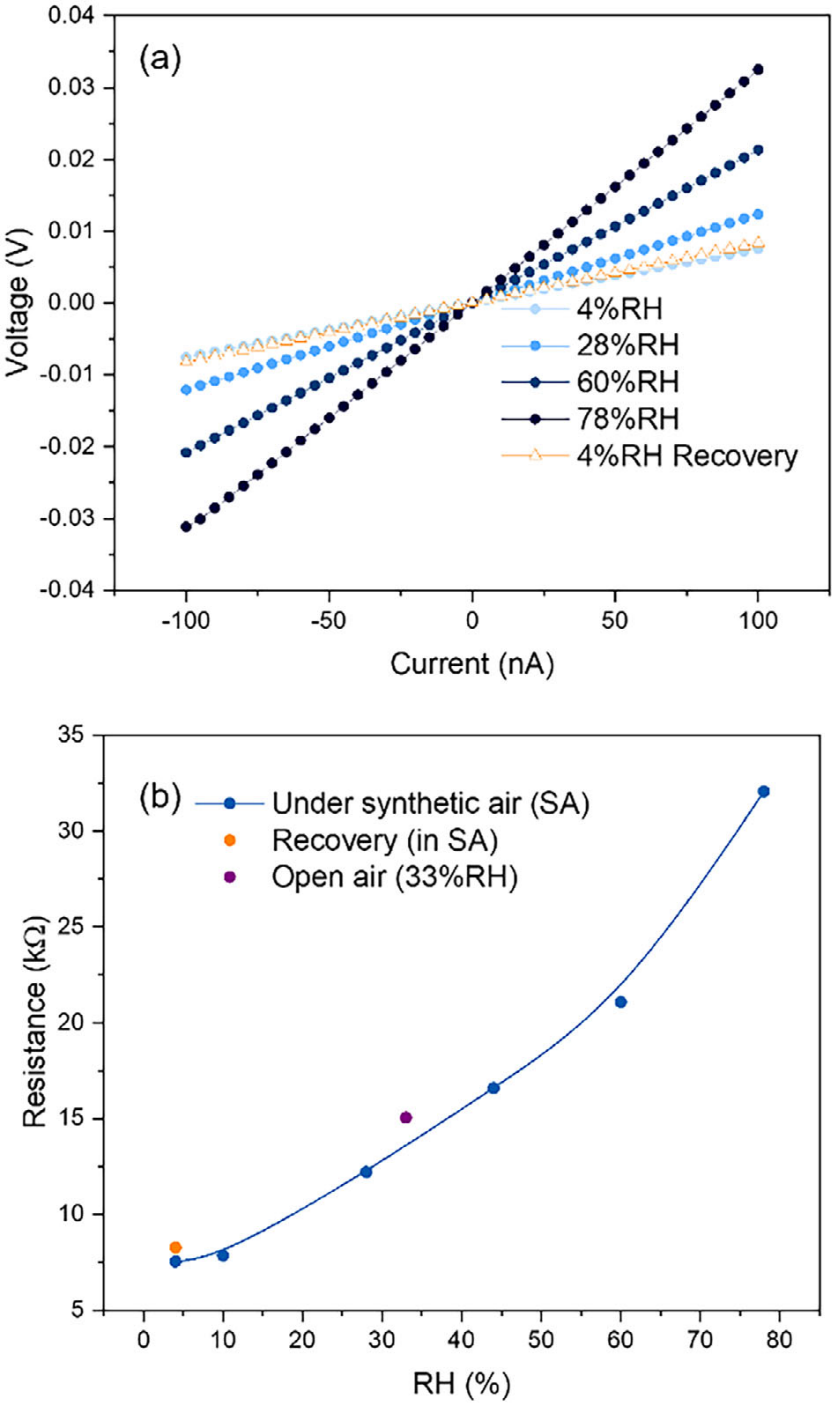
(a) Voltage vs. current curves of Ni‐HHTP deposited on top of the Cr/Au IDE proving the ohmic behavior of the device, under different RH. (b) Corresponding resistance variation. Solid lines correspond to eye guidelines.

The simultaneous increase of the resistance and the enlargement of the crystal lattice of Ni‐HHTP in the presence of increasing RH values, the latter reported in Figure 2, could be understood if an important part of the electrical conduction in Ni‐HHTP occurs along the *c*‐axis, i.e., according to the through‐space pathway mechanism, as the charge carrier mobility would more strongly decrease with increasing layer separation than the extended‐conjugation pathway mechanism [7].

### 2.5 Chemiresistive Properties of Ni‐HHTP in the Presence of CO_2_ and CH_4_

The gas sensing experiments were carried out by measuring the (chemo)resistance variation upon introduction of pulses of varying concentration of the gas of interest, diluted in synthetic air (SA, nominal RH 4%). The measurements were initiated after allowing the device resistance to stabilize in synthetic air at room temperature, ensuring a sufficiently stable baseline.

Then the gas pulses were injected for 5 min, allowing a recovery time of 10 min between pulses. This time was long enough to allow the (almost complete) recovery of the baseline. To monitor and minimize poisoning of the sensor from a previous gas pulse, the injected pulses were applied in a sequence of decreasing concentration over time.

The behavior of the sensor upon pulses of CO_2_, whose concentration ranged between 100 and 800 ppm, is presented in Figure 8a. The presence of CO_2_ produces an increase of the resistance of the device, which is proportional to the concentration of the gas in the atmosphere. As can be seen, the resistance does not reach a steady‐state value at the end of the CO_2_ pulses; however, the recovery—and thus the release of CO_2_—is effective. The chemiresistive response of the device, i.e., the relative variation of the resistance as a function of the CO_2_ concentration, is plotted in Figure 8b. The maximum variation measured for the highest CO_2_ concentration is about 1.6% with a sensitivity of 0.002%/ppm. In a broader context, the response toward CO_2_ observed for Ni‐HHTP falls within the range of chemiresistive performances reported for related materials [24, 36]. In particular, Ni‐complexes derived from other triphenylene‐containing ligands have been reported to exhibit ten times lower sensitivity than Ni‐HHTP, with values on the order of 2 × 10^−4^%/ppm [37] compared to 2 × 10^−3^%/ppm in the present work. An hexaiminobenzene‐based copper material reported by the same authors in the same study [37] exhibited a sensitivity of 4 × 10^−3^%/ppm, which is comparable to the sensitivity reported in the present work.

**FIGURE 8:**
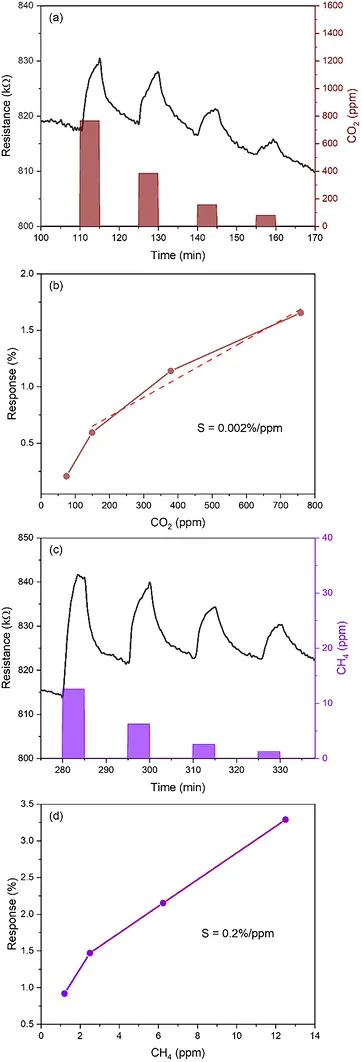
(a) Resistance variation of Ni‐HHTP to different CO_2_ pulses. (b) Corresponding deduced chemiresistive response, with the retrieved sensitivity (S) from a linear regression represented by the dotted line. (c) Resistance variation of Ni‐HHTP with CH_4_ pulses. (d) Deduced chemiresistive response and the obtained sensitivity (S) from a linear regression represented by the dotted line.

Similar experiments were carried out with CH_4_, but with far lower concentrations as for CO_2_, ranging only from 1.25 to 12.5 ppm. For CH_4_, the same trend in chemoresistance as a function of concentration is observed, as shown in Figure 8c: the resistance increases in the presence of the gas. The plot of this increase as a function of CH_4_ concentration is shown in Figure 8d. It is worth noting that the resistance increase can be as large as 3.2% for 12.5 ppm and that even at 1.25 ppm the sensor is able to provide a measurable resistance change. Fitting this change in resistance as a function of concentration in the region displaying the most linear behavior (2.5–12.5 ppm) yields a sensitivity of 0.2%/ppm, with a calculated limit of detection (LOD) of 0.43 ppm of CH_4_. To the best of our knowledge, this is the first published work on the chemiresistive response of Ni‐HHTP (or other triphenylene‐based MOFs) to the presence of CH_4_.

Furthermore, it is worth noting that for both gases the sensor provides a measurable signal at concentration equal or below the actual atmospheric levels of these gases (429.35 ppm for CO_2_ and 1.945 ppm for CH_4_) [38], making this sensor interesting for greenhouse gas monitoring. The retrieved response time of the sensor for CO_2_ is 160 s, whereas a shorter response time of 110 s is obtained for CH_4_. Similarly, a recovery time of 330 s is obtained for CH_4_, which is slightly lower than in the case of CO_2_ (380 s). Overall, the relatively short response and recovery times further postulate Ni‐HHTP as a suitable material for greenhouse gas sensing. Further gas sensing experiments with pulses of varying gas concentrations confirm the reproducibility of the chemiresistive response of the sensor to CO_2_ and CH_4_ (see Figures SI7, SI8 and SI9, respectively).

The higher apparent affinity of Ni‐HHTP for CH_4_ cannot be rationalized by the calculated energetics alone (cfr. previous section on the computational insights into the interactions of Ni‐HHTP with CO_2_ and CH_4_). A more plausible explanation is that the primary CO_2_ binding sites are occupied by water molecules retained from synthesis, which block CO_2_ penetration and restrict its interaction to the surface. In contrast, CH_4_‐accessible sites remain unoccupied even in the presence of water, resulting in a higher apparent response to methane. In summary, the adsorption selectivity of Ni‐HHTP reflects the interplay between electrostatic potentials and the structural constraints of the lattice. Although fragment‐based models predict strong H_2_O and CO_2_ binding, the crystal packing renders many of these sites inaccessible, and their occupation by residual water further limits CO_2_ uptake. By contrast, open channels provide adsorption pockets that can accommodate CH_4_, whose weaker interactions are nevertheless unhindered. Thus, the observed preference for methane arises not from stronger binding, but from its ability to access the available sites within the hydrated framework.

## 3 Conclusions

In this study, we report the crystal structure of Ni‐HHTP, resolved from single‐crystal electron diffraction data. The structure comprises two alternating layers based on the Ni_3_(HHTP)_2_·6H_2_O and Ni_3_(HHTP)_1_·12H_2_O cores. The former forms an extended two‐dimensional framework featuring radical semiquinonate coordination, whereas the latter consists of a two‐dimensional layer via hydrogen‐bonded Ni_3_(HHTP)_1_·12H_2_O moieties. The overall crystal architecture alternates between radical‐containing and closed‐shell HHTP‐based layers along the crystallographic *c*‐axis. In both fragments, water molecules occupy all available coordination sites of the nickel centers, completing an octahedral geometry. The Ni‐HHTP crystal structure is responsive to changes in relative humidity, causing a swelling of the crystal lattice, with a larger effect on the *c*‐axis. This process is fully reversible, indicating that reversible adsorption‐desorption of water molecules occurs within the framework.

The current‐voltage characterization of Ni‐HHTP powder between interdigitated Au electrodes shows an ohmic behavior, whose resistance increases with relative humidity concentration. This result can be interpreted by assuming a significant contribution of the through‐pathway mechanism to the conduction. The exposure to CO_2_ and CH_4_ gives also rise to increase of resistance, allowing the detection of concentrations as low as 100 ppm of CO_2_ and 1.25 ppm of CH_4_, which makes this material suitable for its use as greenhouse gas sensors.

Computational studies show that the binding energy of H_2_O to Ni‐HHTP is markedly higher than those of CO_2_ and CH_4_. While CO_2_ and H_2_O share similar preferred binding sites, CH_4_ binds at distinct locations within the framework. This difference may account for the higher sensitivity of Ni‐HHTP toward CH_4_: water molecules occupying the strong CO_2_/H_2_O sites hinder CO_2_–Ni‐HHTP interactions, whereas CH_4_ can access its binding pockets more freely, resulting in a higher chemiresistive response.

## Conflicts of Interest

The authors declare no conflicts of interest.

## Supporting information




**Supporting File**: smll75151‐sup‐0001‐SuppMat.pdf.

## Data Availability

The data that support the findings of this study are available in the supplementary material of this article.
