# Supplementary material for: Layered Nickel–HHTP Networks for Efficient Chemiresistive Sensing of CH4 and CO2
Source: Small. 2026 Aug 22;22(58):e00029. doi: 10.1002/smll.202600029 (PMC13676835; doi:10.1002/smll.202600029)
Supplement: Supplementary file 1 [file SMLL-22-e00029-s001.pdf]

Supporting Information

Layered Nickel-HHTP Networks for Efficient Chemiresistive Sensing of CH_4 an CO_2

Ignasi Fort-Grandas, Marta Martínez-Belmonte, Jordi Benet-Buchholz, Rosa M. Gomila, Antoni Frontera*, Albert Romano-Rodríguez,* and Anton Vidal-Ferran*

Contents

1.-	Materials, methods, statistical analysis and synthetic protocols	2
2.-	Imaging, elemental composition and characterization of Ni-HHTP crystals	4
3.-	Assignment of diffraction peaks of Ni-HHTP	6
4.-	Humidity effects on Ni-HHTP	8
5.-	Electrical Conductivity Measurements	9
6.-	Gas Sensing Measurements	10
7.-	Crystallographic Details	13
8.-	Computational Studies	30
9.-	Cartesian coordinates	31

1.- Materials, Synthetic Protocols and Methods

Materials

All commercially available chemicals were used without further purification unless otherwise noted.

Methods

<u>Powder X-ray diffraction (pXRD)</u>

X-ray powder diffraction analyses were carried out using Cu K-alpha radiation in a Malvern-Panalytical Empyrean Diffraction Device configured in Omega/2Theta geometry and equipped with a PIXcel1D-Medipix3 detector and an Empyrean Cu LFF HR Xray Tube at 45 kV 40 mA. Studies of variable temperature and humidity were carried out in an Anton Paar CHC plus+ Cryo & Humidity Chamber with an MHG Humidity Generator. Measurement parameters: 4-40 2theta deg, 0.013deg/s, 100s/step.

Working conditions:

- Variable Relative Humidity (RH): ramping from 10% to 80% RH, then returning to 10% at 25 ºC.
- Variable Temperature: ramping from 10 ºC to 80 ºC, then returning to 10 ºC at 20% and 80% RH.

<u>Field-Emission Scanning Electron Microscopy (FESEM) and Energy dispersive X-ray Spectroscopy (EDS)</u>

Scanning Electron Microscopy was carried out in a JEOL JSM 7100-F microscopy, equipped with a field emission gun and operating between 5 and 20 kV. The images, formed by the emitted secondary electrons, have been acquired employing an Everhart–Thornley detector. The machine is also equipped with a Energy Dispersive GATAN ALTO-1000 spectrometer, that allows the detection of elements with atomic number above 4.

<u>Nitrogen adsorption measurements</u>

Nitrogen adsorption–desorption measurements were performed using a Micromeritics Tristar 3000 analyzer. The measurements were conducted at relative pressures (P/P_0) of 0.01, 0.05, 0.10, 0.15, and 0.20.

Statistical Analysis

I-V curves were performed as a current sweep, from – 100 nA to 100 nA, consisting of 40 points, each one being the average of 3 measurements. Gas sensing results are reported from untreated raw data unless stated otherwise in the figure caption. For samples where signal drift is removed, baseline adjustment and subtraction has been performed by means of a Spline interpolation method. Each gas sensing figure corresponds to a separate Ni-HHTP sensor, fabricated with MOF samples coming from different batches to ensure reproducibility. To evaluate sensor response, response times and recovery times, the response was taken as the rise from the 10% to 90% as is one of the usual ways employed in sensor measurements and in electronics in general. Sensitivity values, for both CH_4 and CO_2 gas responses, were calculated by linear regression of the sensor response at different gas concentrations. The response from lowest pulse concentration was excluded for sensitivity calculations due to possible limitations in gas flow mixture of the setup. The LOD values are calculated using the standard formula of LOD = 3*σ/S. OriginLab software was used for all data treatment and calculations.

Synthetic protocols: Synthesis of Ni-HHTP

Ni-HHTP was synthesized following a solvothermal method. 9.6 mg of nickel acetate tetrahydrate (0.038 mmol) were dissolved in 1.5 mL of Mili-Q ultrapure water in a 15 mL glass vial. Then, 7 mg of triphenylene-2,3,6,7,10,11-hexaol (HHTP, 0.022 mmol) were added at once and the vial was sonicated for 10 min. Afterwards, 0.15 mL of DMF were added dropwise (0.1 mL/min). The mixture was sonicated for 10 more min. Then, the capped vial was put in the oven at 80ºC for 6 h. The resulting suspension was centrifuged and washed with water (3 x 8 mL), EtOH (3 x 8 mL) and dried under vacuum to obtain 3.5 mg (yield 33%) of a dark powder.

The yield is calculated to a 1:1 molar mixture of $Ni_3(HHTP)_2 \cdot 6H_2O$ and $Ni_3(HHTP) \cdot 12H_2O$, giving a general formula of $Ni_6(HHTP)_3 \cdot 18H_2O$.

2.- Imaging, Elemental Composition and Characterization of Ni-HHTP Crystals

Images of the crystals were taken with Field Emission Scanning Electron Microscopy (FESEM) and their elemental composition was determined with Energy-Dispersive X-ray Spectroscopy (EDS).

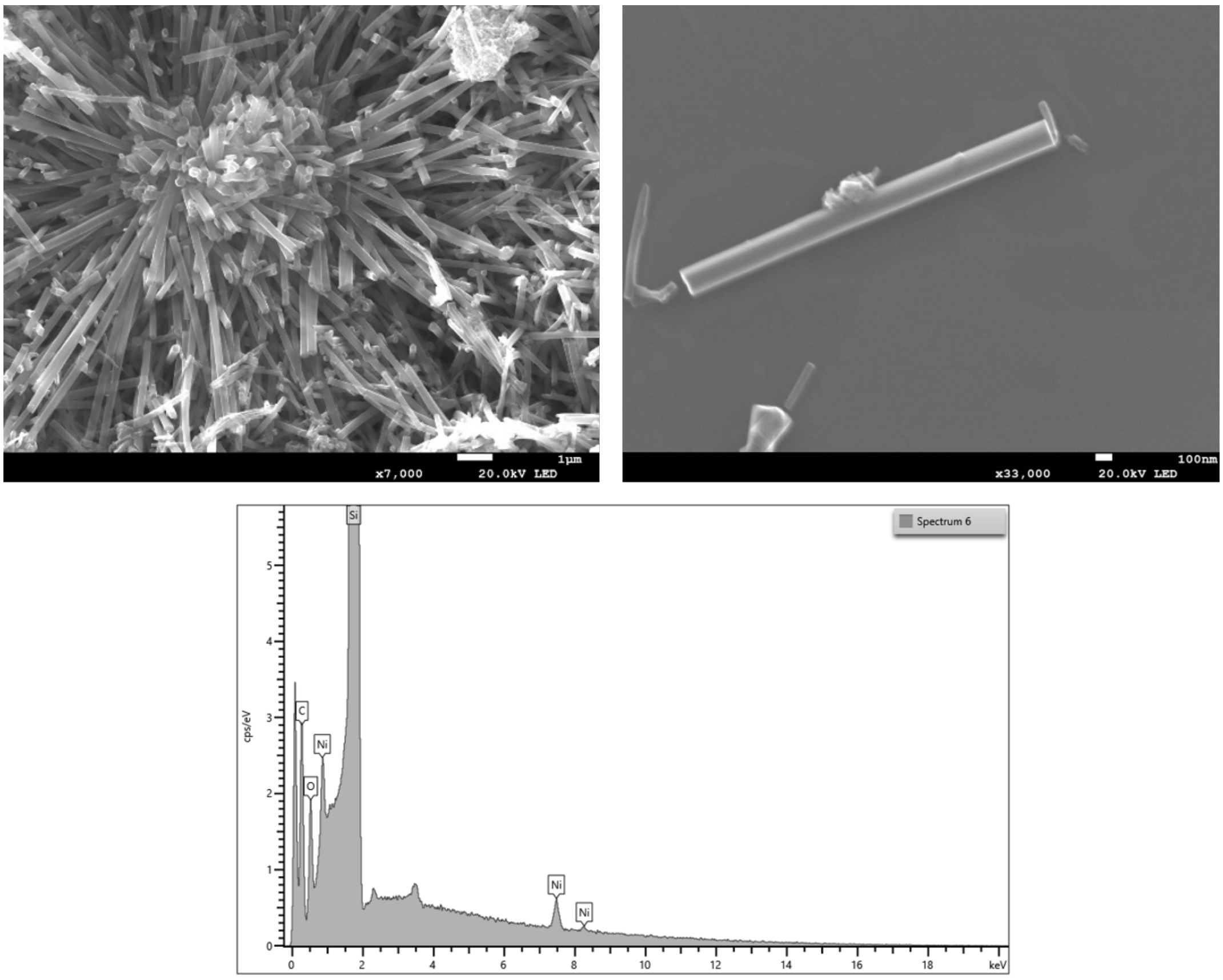

Figure SI1. FESEM images of aggregated Ni-HHTP and single particles of Ni-HHTP with the corresponding EDS spectrum

The FESEM images reveal a well-defined rod-like morphology for the Ni-HHTP particles. This morphology remains consistent and homogeneous in the aggregated MOF particles, as shown in the corresponding FESEM image. The rods typically measure approximately 10 µm in length and 300 nm in diameter. EDS analysis confirms the expected elemental composition of the MOF, indicating the presence of C, O, and Ni. The detected Si signal originates from the substrate on which the MOF powder was deposited for SEM imaging. The sulfur signal likely originates from trace impurities present in the reagents or solvents used during synthesis.

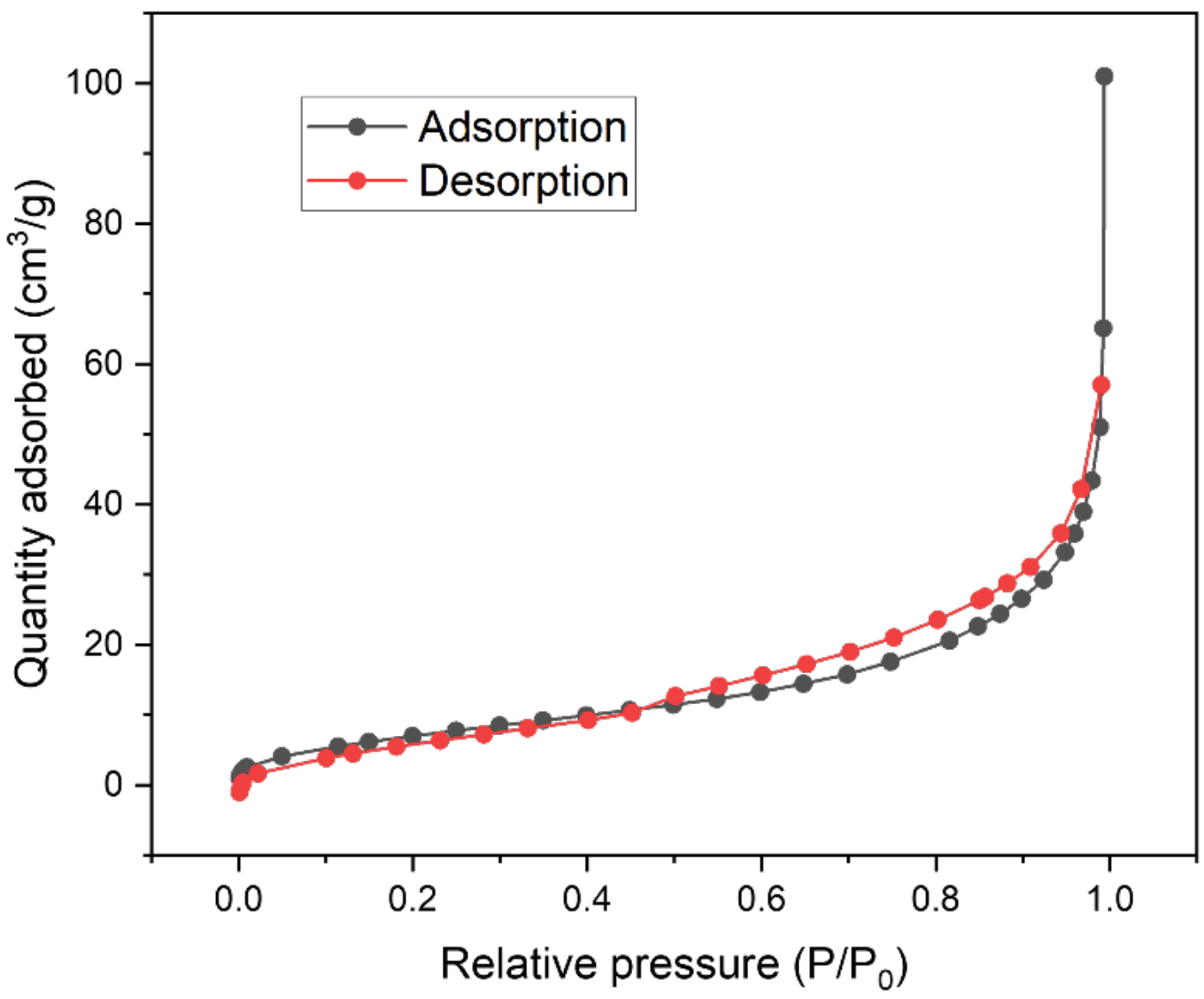

Figure SI2. Nitrogen adsorption-desorption isotherms at 77 K.

The BET surface area of Ni-HHTP was determined to be 28.73 ± 0.15 $m^2\ g^{-1}$, with micropore volumes of 15 $m^3\ g^{-1}$. The micropore volume is consistent with analogous HHTP-based materials synthesized by other groups[1], whereas the smaller surface area is attributed to the MOF being prepared analogously to the drop casting procedure used for sensing experiments, i.e. without performing the thermal activation process described in reference 1.

[1] M. Hmadeh, Z. Lu, Z. Liu, F. Gándara, H. Furukawa, S. Wan, V. Augustyn, R. Chang, L. Liao, F. Zhou, E. Perre, V. Ozolins, K. Suenaga, X. Duan, B. Dunn, Y. Yamamto, O. Terasaki and O. M. Yaghi, "New Porous Crystals of Extended Metal-Catecholates," *Chem. Mat.* **2012**, *24*, 3511-3513.

3.- Assignment of Diffraction Peaks of Ni-HHTP

To characterize the powder diffraction pattern of Ni-HHTP and assign the corresponding Miller indices, single-crystal data were used to simulate the theoretical pattern using the Mercury software package.[2]

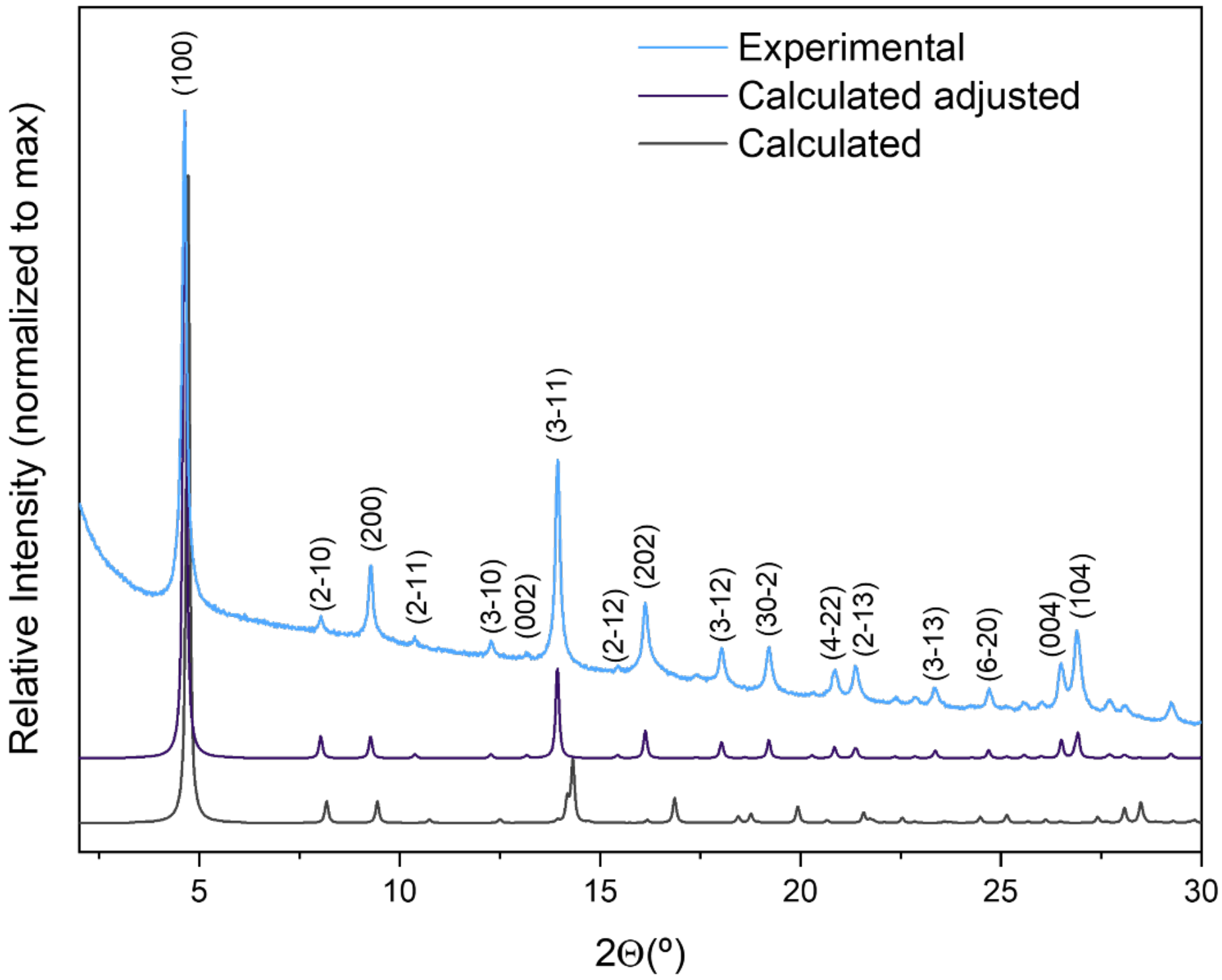

Figure SI3. Experimental PXRD (298.15 K and 65% RH) of Ni-HHTP (blue graph) with the Miller indexes assigned to the diffraction peaks. Calculated diffraction pattern at 100 K and unknown water content with the Mercury Software Package[2] from the single crystal structure (grey graph). Calculated PXRD pattern (purple graph) employing lattice parameters *a* and *c* adjusted to 25 ºC and 65% RH

Single-crystal samples for electron-diffraction data acquisition were initially cryo-prepared and measured at 100 K under vacuum, whereas the experimental PXRD was recorded at 298.15 K and 65 % RH. The resulting shift in peak positions in PXRD is consistent with the humidity-induced effect described in this work, in which increased humidity causes diffraction peaks to shift toward lower angles. To simulate the PXRD under the experimental recording conditions (298.15 K and

---

[2] C. F. Macrae, I. Sovago, S. J. Cottrell, P. T. A. Galek, P. McCabe, E. Pidcock, M. Platings, G. P. Shields, J. S. Stevens, M. Towler, P. A. Wood, "Mercury 4.02: From Visualization to Analysis, Design and Prediction", *J. Appl. Crystallogr.* **2020**, *53*, 226-235.

65 % RH), rather than under those of the electron diffraction measurements (100 K and unknown RH), an adjustment of the *a* and *c* lattice parameters was performed. The interplanar spacings $d_{(004)}$ and $d_{(100)}$ were calculated from their corresponding experimental diffraction peaks at 298.15 K and 65 % RH using Bragg's law. The $d_{(004)}$ and $d_{(100)}$ values at 298.15 K and 65 % RH were then used to calculate corrected lattice parameters *a* and *c* at the new experimental conditions by applying the formulae in Eq. 1, which derive from the general formula in Eq. 2 for interplanar spacings in a trigonal crystal system.[3] The resulting parameters (*a* = 22.03, *c* = 13.45 Å at 298.15 K and 65 % RH) differ from those obtained from electron diffraction at 100 K and unknown RH (*a* = 21.62(14) Å, *c* = 12.70(19) Å). These adjusted *a* and *c* lattice parameters were subsequently employed to simulate the PXRD pattern at 298.15 K and 65 % RH using the Mercury software package,[2] while all remaining crystallographic parameters required for simulating the PXRD pattern at the new experimental conditions were retained as determined from electron diffraction.

$$a = d_{(100)}\sqrt{\frac{4}{3}} \qquad c = 4d_{(004)} \qquad \text{Eq. 1}$$

$$\frac{1}{d^2} = \frac{4(h^2+hk+k^2)}{3a^2} + \frac{l^2}{c^2} \qquad \text{Eq. 2}$$

The diffraction peaks in Figure SI3 show good agreement between the experimental data and the pattern calculated from adjusted *a* and *c* lattice parameters at 298.15 K and 65 % RH. The close match in both peak positions and intensities enables the assignment of most reflections in the 2θ range from 2° to 30° to the corresponding Miller indices.

---

[3] R. Ubic, *Crystallography and Crystal Chemistry*, 1st ed., Springer Cham, **2024**.

4.- Humidity effects on Ni-HHTP

The sample was placed in the sample holder in the CHC plus+ chamber. Powder X-ray diffraction studies were carried out with *in situ* temperature and relative humidity-controlled conditions. All studies began at 25°C and 20% RH, after which different series of temperature or humidity variations were applied. Figure SI4 presents the powder diffraction patterns recorded at 25 °C, in which the relative humidity was systematically varied from 10% to 80% RH in 10% increments. The ramp-up from 10% to 80% RH is shown in Figure SI4a whereas the subsequent ramp-down to lower RH is shown in Figure SI4b. This measurement corresponds to an extension of the experiment reported in Figure 2 of the manuscript.

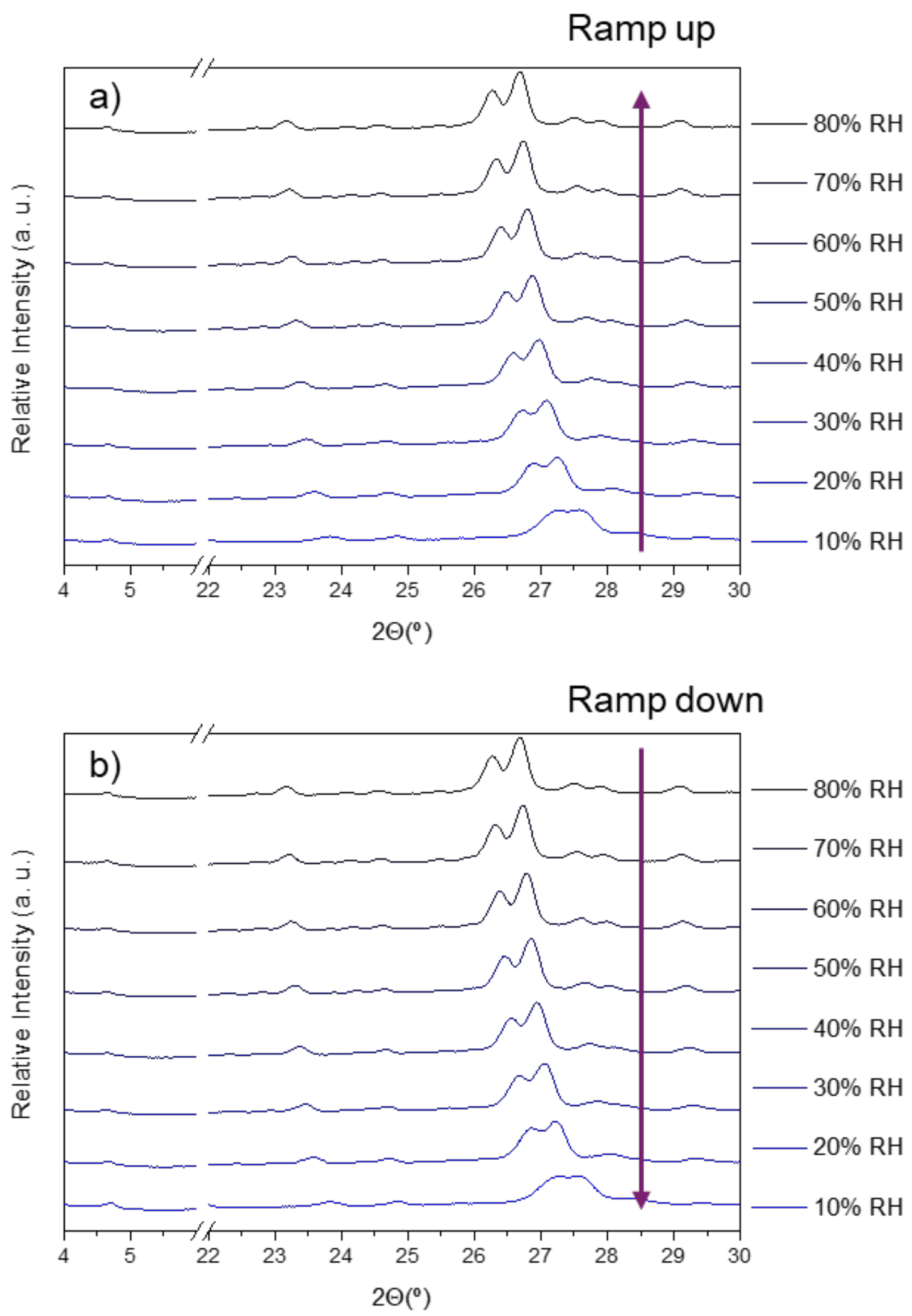

Figure SI4. a) PXRD of Ni-HHTP at 25ºC with humidity increasing from 10% RH to 80% RH. b) Corresponding ramp down in humidity from 80% RH back to 10% RH.

5.- Electrical Conductivity Measurements

An electronic chip consisting of Au electrodes on fused silica was used for the electrical conductivity measurements. The electrodes were fabricated using UV-photolithography, depositing Cr/Au 10/150 nm-thick metal employing electron-beam evaporation, followed by lift-off in Remover 1165, that dissolves the remaining photoresist. The mask used in the photolithography gave rise to an interdigitated (IDE) shape, where the electrode's width and the interdigitate distance were both of either 10 or 50 µm. The chip was mounted on top of a TO-8 support and the electrodes of the chip were wire-bonded with Au to the pins of the TO-8. To perform the electrical measurements, the TO-8 was connected to a Keithley 2600 Source Measure Unit, that allows the simultaneous electrode bias and current measure. Figure SI5 shows an image of the chip, with the enlargement of the IDE area. In this case the IDE spacing and width is 50 µm.

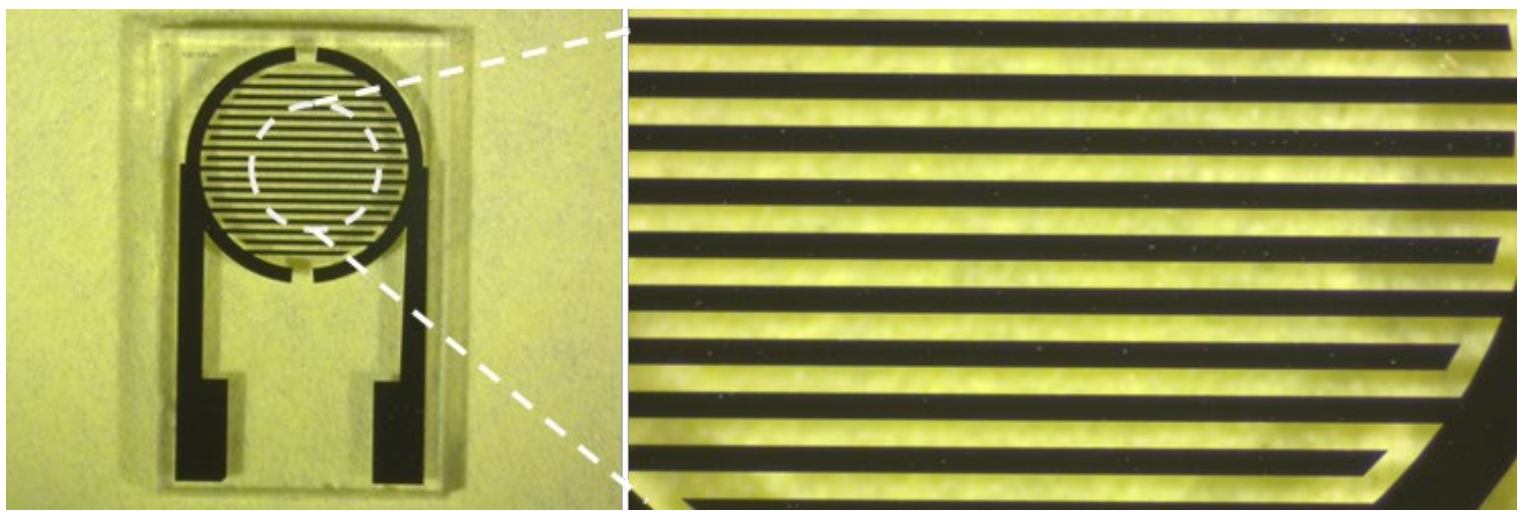

Figure SI5. Optical images of the IDE Chips with the interdigitated region with the deposited MOF.

The sample preparation for its dispersion on the IDE is as follows: a fresh suspension of 2 mg/mL of Ni-HHTP in Millipore water was sonicated for 5 min. From this suspension, 1.5 µL were drop-casted onto the interdigitate region of the chip, and dried at 80ºC for 15 min.

6.- Gas Sensing Measurements

The gas sensing setup is as follows. The IDE with the deposited Ni-HHTP material was introduced in a sealed chamber of the gas sensing setup and directly used as a sensor. A schematic of the setup is provided in Figure SI6.

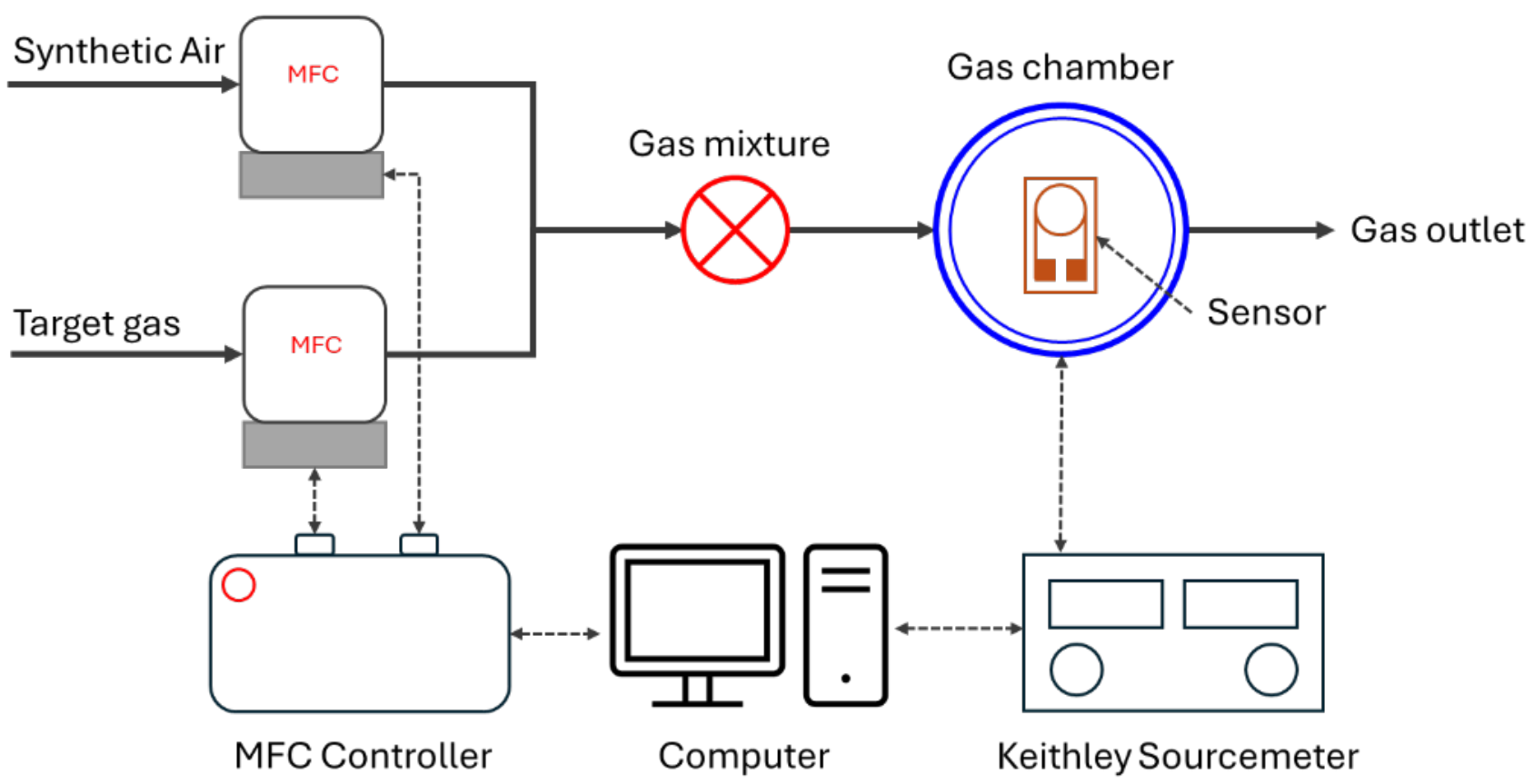

Figure SI6. Schematic representation of the setup used to perform electrical and gas sensing measurements. MFC stands for Mass-Flow-Controller.

Chemiresistive gas sensing experiments were performed after electrical characterization of the samples. Humidity levels were lowered to 4% RH by flowing dry synthetic air (SA), and the samples were aged for hours in order to stabilize as much as possible the drift in the baseline resistance. Afterwards, the sensors were exposed to pulses CO_2 or CH_4 by mixing of SA and the corresponding target gas, while always keeping the total flux of system constant.

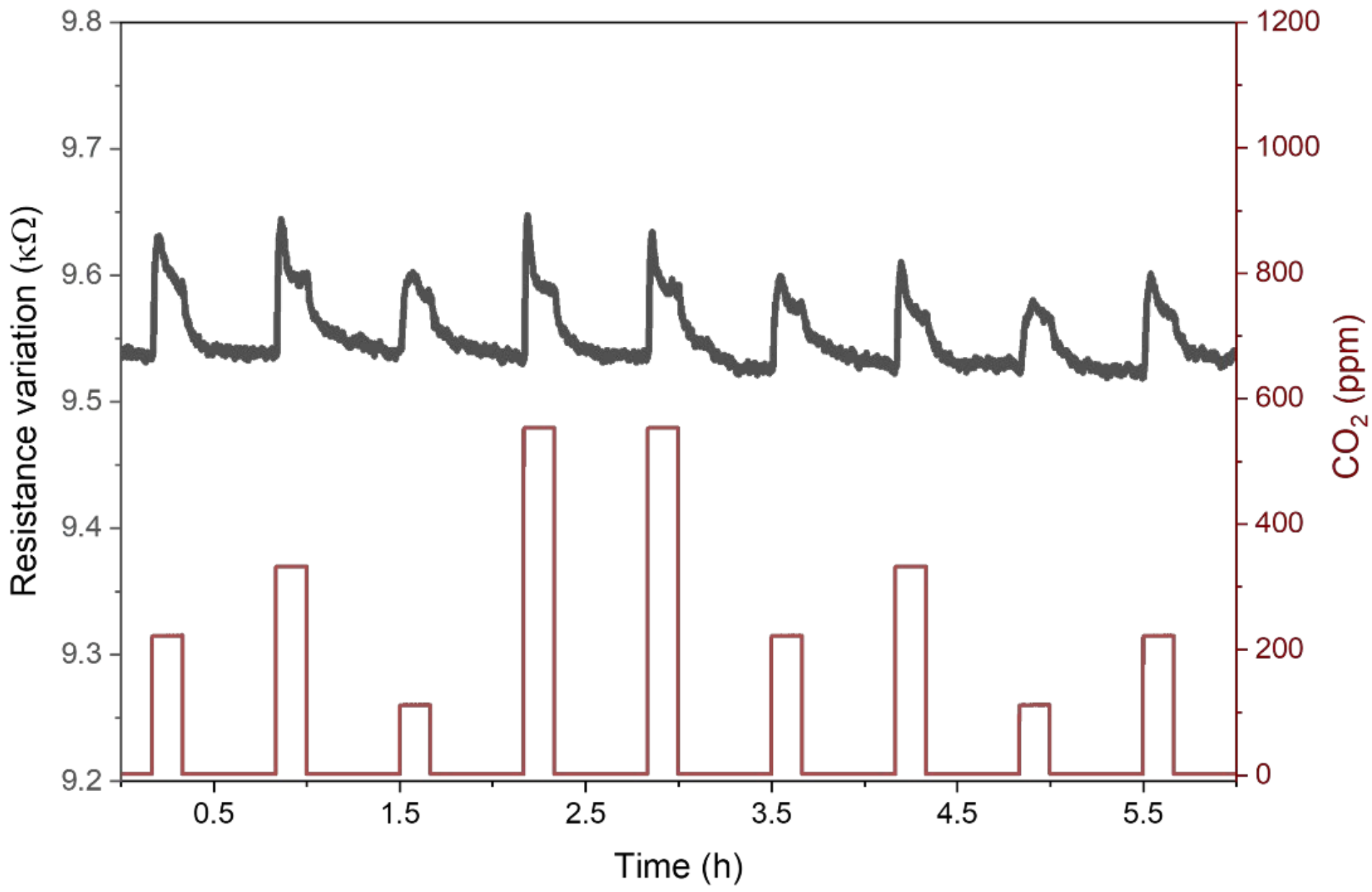

Figure SI7. Resistance variation of Ni-HHTP MOF towards CO_2 pulses of varying concentrations. Signal drift has been subtracted.

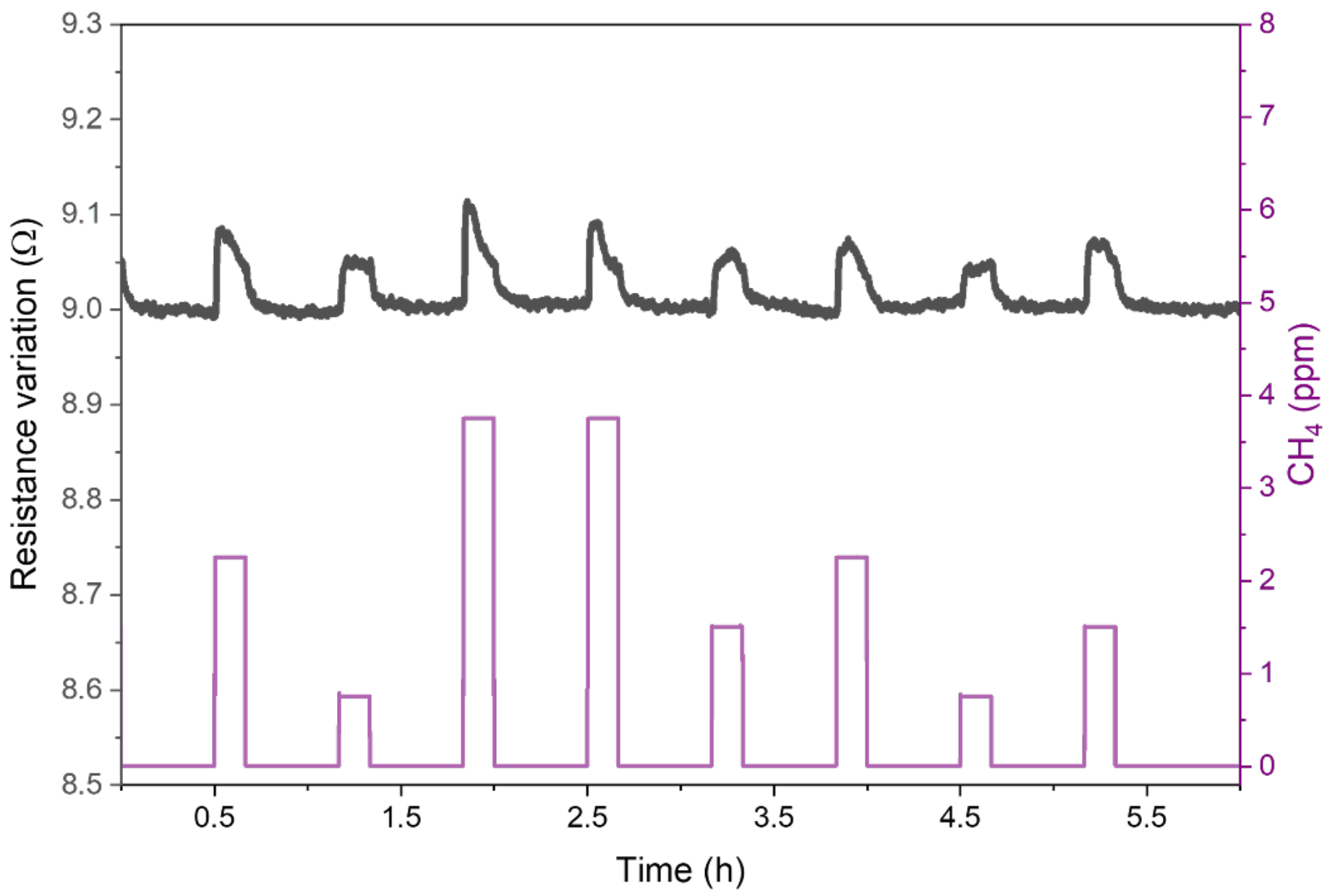

Figure SI8. Resistance variation of Ni-HHTP MOF towards CH_4 pulses of varying concentrations. Signal drift has been subtracted.

The sensors show a consistent chemiresistive response to pulses of CO_2 and CH_4 gases under dry synthetic air and room temperature conditions. The

responses shown in Figures SI7 and SI8 have been obtained at concentration below atmospheric concentrations for both CO_2 and CH_4, proving the suitability of these sensors for practical GHG detection. In order to assess the reproducibility, four sensors were fabricated using material from two different Ni-HHTP synthetic batches. Measurements were performed over a period of one month. The dark red line in Figure SI9 is the average response as a function of time, while the grey represents the standard deviation of these measurements, confirming the reproducibility of the response of the sensors.

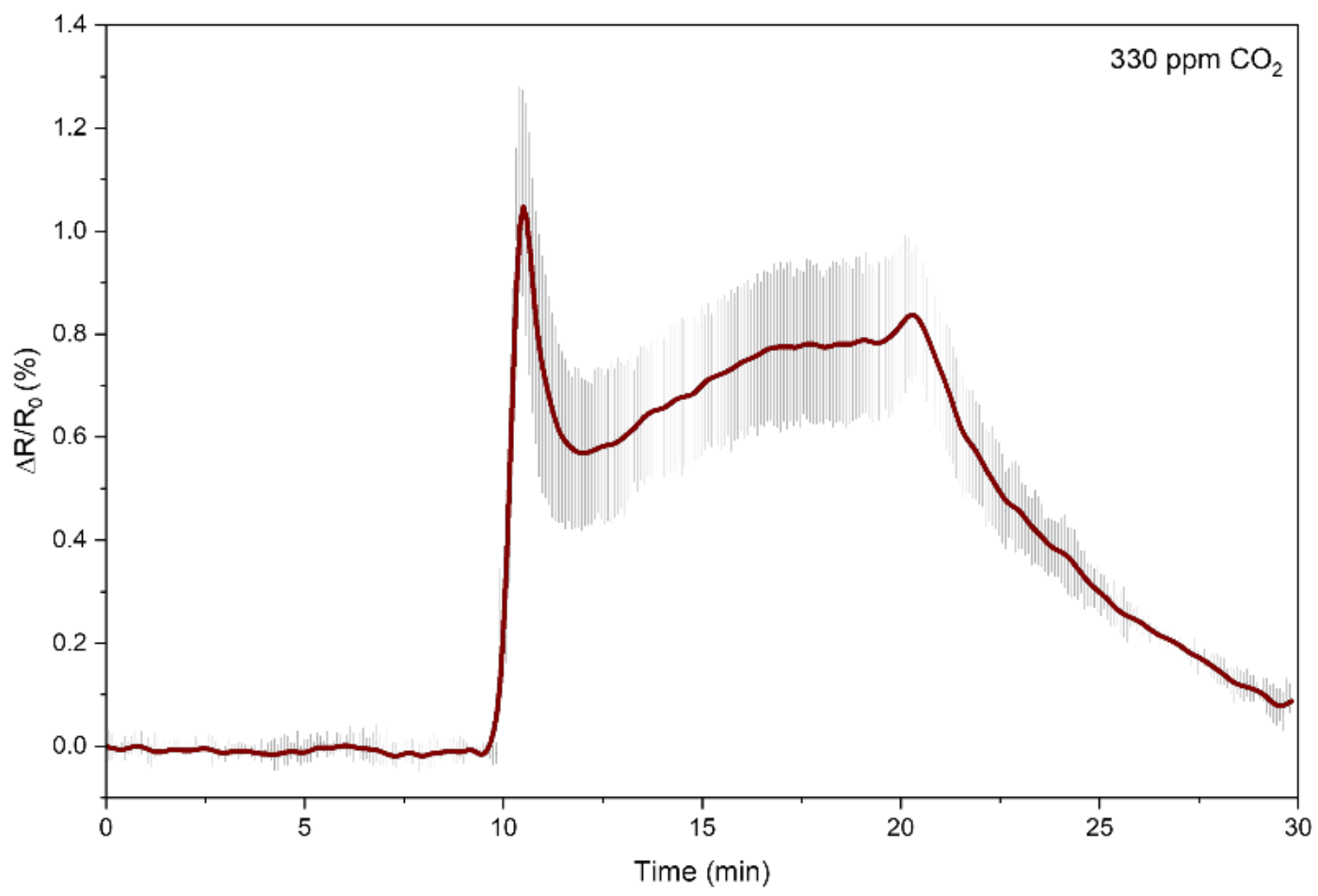

Figure SI9. Average response of 4 sensors (in dark red) to pulses of 330 ppm of CO_2, with standard deviation in grey.

7.- Crystallographic Details

Sample preparation

The sample was prepared on a copper-graphite grid (Lacey C only 200 TH Cu TED PELLA INC.) by inserting the grid inside the original vial and shaking together with the pulverized sample. The excess amount of sample was removed by knocking carefully with the clamp on the supporting slide.

Cryo-preparation

The grid with the sample underwent cryo-preparation using an ELSA sample holder and the cryo-preparation stage from GATAN. In order to avoid ice formation, the sample was prepared at 215 K and mounted in the specimen chamber. The sample was hold for 15 minutes at 215 K to sublimate water crystals and cooled down to 100 K. The measurements were performed at 100 K (LT).

Device

The sample grains were measured using a XtalLAB Synergy-ED from Rigaku-JEOL provided with a HyPix-ED detector and a LiB6 200 kV electron source (200 kV, 101.20 μA) in shutterless operation mode.[4] Wavelength: 0.0251 Å. Total dose for the three merged crystal grains: 22.618 $e^{-}/Å^{2}$, condenser strength of 1. Magnification diffraction: 50 cm, IL1 projection focus: hex 52C1 (21185), CL 10 μm, SA 100 μm, distance (camera length): 644 mm, scan width: 0.25° and exposure time was depending on the crystal in the range 0.25-0.50 s/degree.

Accessories

ELSA (GATAN) sample holder and sample preparation stage for preparation and measurement of samples at low temperatures.

[4] S. Ito, F. J. White, E. Okunishi, Y. Aoyama, A. Yamano, H. Sato, J. D. Ferrara, M. Jasnowski, M. Meyer, "Structure Determination of Small Molecule Compounds by an Electron Diffractometer for 3D ED/MicroED", *CrystEngComm* **2021**, *23*, 8622–8630.

Software

Measurement and data processing: CrysAlis^{Pro} 43.120a (Rigaku)[5]. Structure processing: Olex2 Version 1.5-ac6-019 © OlexSys Ltd. 2004 – 2024[6]. Dynamical structure refinement: Jana2020 Version 1.3.53, 19/01/2024.[7]

Measurement and refinement[8]

The samples Ni-HHTP were explored on the XTalLAB Synergy-ED at 100 K.

- 11 crystals were measured at LT from which 9 could be indexed and gave the same trigonal cell.

Initial indexing was performed with the AutoChem 6[9] software system in combination with Olex 2 giving a trigonal cell with the space group *P*-3c1 (No. 165). No other crystalline phases could be detected in the crystals explored. Unit cell parameters are shown in Table SI1.

Table SI1. Trigonal unit cell (*P*-3c1) indexed at 100 K for all selected grains.

	Unit cell parameters						Volume
Grain	A (Å)	B (Å)	C (Å)	α (°)	β (°)	γ (°)	(Å^3)
07	21.62(14)	21.62(14)	12.70(19)	90	90	120	5137(89)
09	21.63(10)	21.63(10)	12.71(14)	90	90	120	5152(67)
05	21.66(9)	21.66(9)	12.69(12)	90	90	120	5155(59)

[5] Rigaku Oxford Diffraction, CrysAlis^{Pro} Software System, Rigaku Corporation, Wrocław, Poland, **2023** (version 1.171.43.53a).

[6] O. V. Dolomanov, L. J. Bourhis, R. J. Gildea, J. A. K. Howard, H. Puschmann, "OLEX2: A Complete Structure Solution, Refinement and Analysis Program", *J. Appl. Cryst.* **2009**, *42*, 339–341.

[7] V. Petříček, L. Palatinus, J. Plášil, M. Dušek, "Jana2020 – a New Version of the Crystallographic Computing System Jana", *Z. Kristallogr. Cryst. Mater.* **2023**, *238*, 271-282.

[8] Crystallographic data for the structure(s) reported in this paper have been deposited with the Cambridge Crystallographic Data Centre (CCDC) under deposition number [2488168]. These data can be obtained free of charge from the CCDC via www.ccdc.cam.ac.uk/data_request/cif.

[9] Rigaku Oxford Diffraction, AutoChem 6 Software System in Conjunction with OLEX2, Rigaku Corporation, Wrocław, Poland, **2023** (version 1.5-ac6-009).

A structure solution was successfully obtained from several crystals measured at 100 K. One of the best datasets measured corresponded to crystal 07 which was used for structure solution using the program ShelXT.[10] (see Tables SI5-SI8 for structure refinement results, R1: 15.56 % and Completeness: 99.9 %). The instrument was operated, and the diffraction data were processed in the program CrysAlis^{Pro}. Absorption correction was performed using spherical harmonics implemented in SCALE3 ABSPACK scaling algorithm in CrysAlis^{Pro}. To enhance the reflections-to-parameter ratio and reduce the dynamic effects, the datasets of the 3 best selected crystals were merged resulting in a single improved data set [Crystals: 07, 09 and 05]. By merging data, a completeness of 100 % up to a resolution of 0.80 Å was achieved. An overview of data collection parameter and quality statistics for all single data collections is show in Table SI2 and Table SI3. Images and selected frames of the measured crystals are shown in Figure SI10.

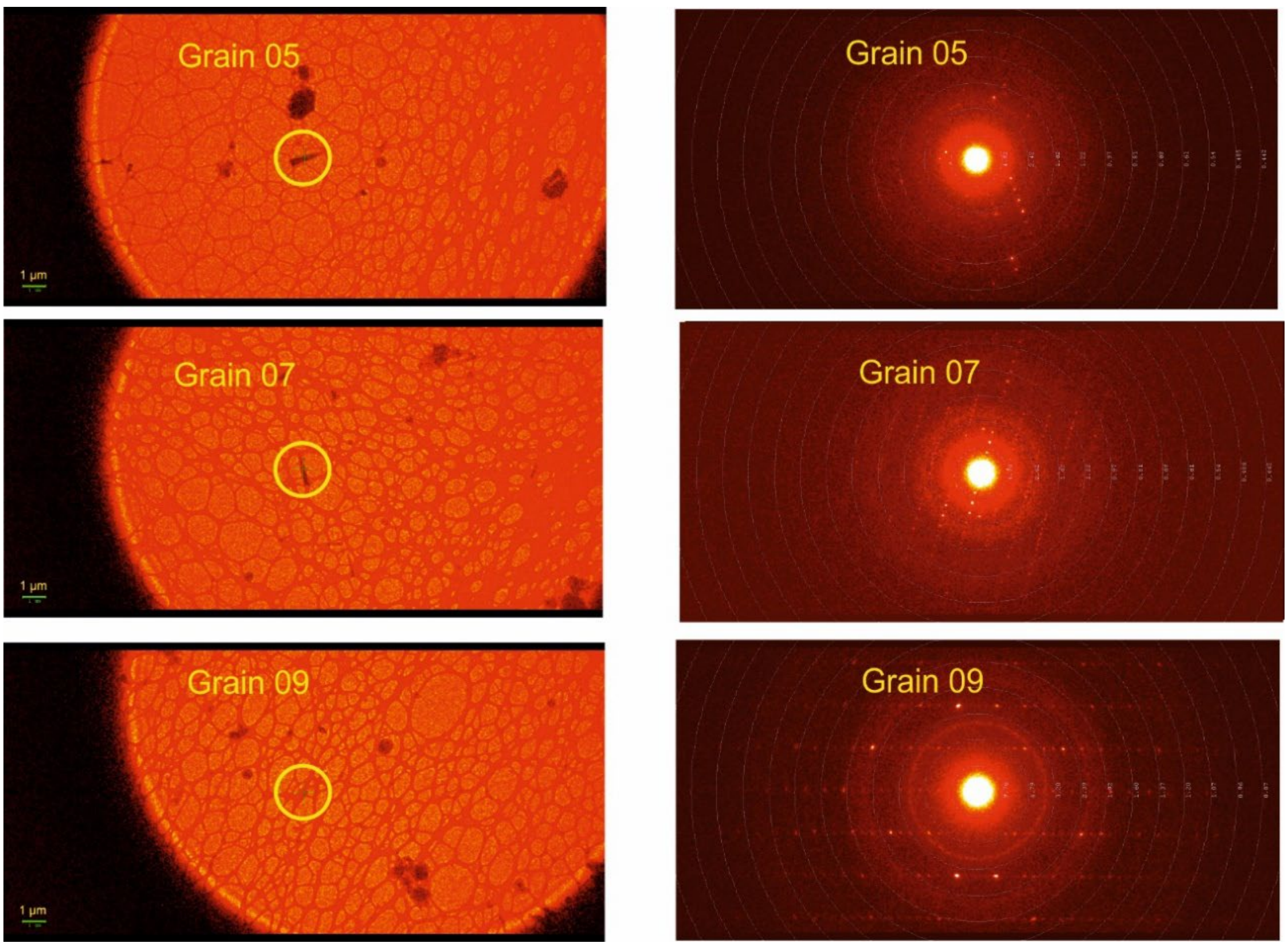

Figure SI10. Grain snapshots and chosen diffraction images for all selected measurements. Grains 05, 07 and 09.

[10] Program for structure solution XT: G. Sheldrick, "SHELXT - Integrated Space-group and Crystal-structure Determination", *Acta Crystallogr. A*. **2015**, *71*, 3-8.

The merged dataset was used for structure refinement with a kinematical approximation using ShelXL[11] in the crystallographic program suite OLEX2. The structure refined to a final R1-value of 14.98 % (see Table SI4 for overview and Tables SI9 to SI12). An ORTEP-plot drawing showing the asymmetric unit for structure Ni-HHTP is represented in Figure SI11. The asymmetric unit contains two different nickel atoms coordinated to the ligand molecules forming two independent sub-structures. Additionally, there is a volume which contains 1.4 water molecules that are disordered in 5 positions and are interacting through hydrogen bond with the water molecules coordinated to the metal atom. These water molecules are relatively diffuse and form a void in their central area. One of the nickel atom type (Ni2, Fragment A) is coordinated to two ligands and two water molecules. The second nickel atom type (Ni1, Fragment B) is coordinated to one ligand and four water molecules. In the case of Ni2 (Fragment A), by growing the structure based on the symmetry, a two-dimensional polymer network is formed (see Figures SI12 and SI13). In the case of Ni1 (Fragment B) single molecules are formed which interact with other molecules through hydrogen bonds of the water molecules (see Figure SI14). The two different fragments generated by Ni1 and Ni2 are interacting through π-interactions of the aromatic rings and hydrogen bonds of the water molecules coordinated to Ni, forming a sandwich like arrangement (see Figure SI15), yielding the whole MOF crystalline structure represented in Figure SI16. Non-hydrogen atoms were assigned anisotropic displacement parameters unless stated otherwise. The hydrogen atoms were placed in idealized positions and included as riding. Isotropic displacement parameters for all H atoms were constrained to multiples of the equivalent displacement parameters of their parent atoms with $U_{iso}(H)$ = 1.2 (rigid H-atoms) or 1.5 (hydrogen atoms belonging to -OH) U_{eq}(parent atom). Enhanced rigid bond restraints,[12,13] with standard uncertainties in the range of 0.01-0.004 $Å^2$ were applied.

---

[11] G. M. Sheldrick, “A Short History of SHELX”, *Acta Crystallogr. A.* **2008**, *64*, 112-122.

[12] F. Hirshfeld, "Can X-Ray Data Distinguish Bonding Effects from Vibrational Smearing?", *Acta Crystallogr. A.* **1976**, *32*, 239-244.

[13] A. Thorn, B. Dittrich, G. M. Sheldrick, "Enhanced Rigid-Bond Restraints", *Acta Crystallogr. A.* **2012**, *68*, 448-451.

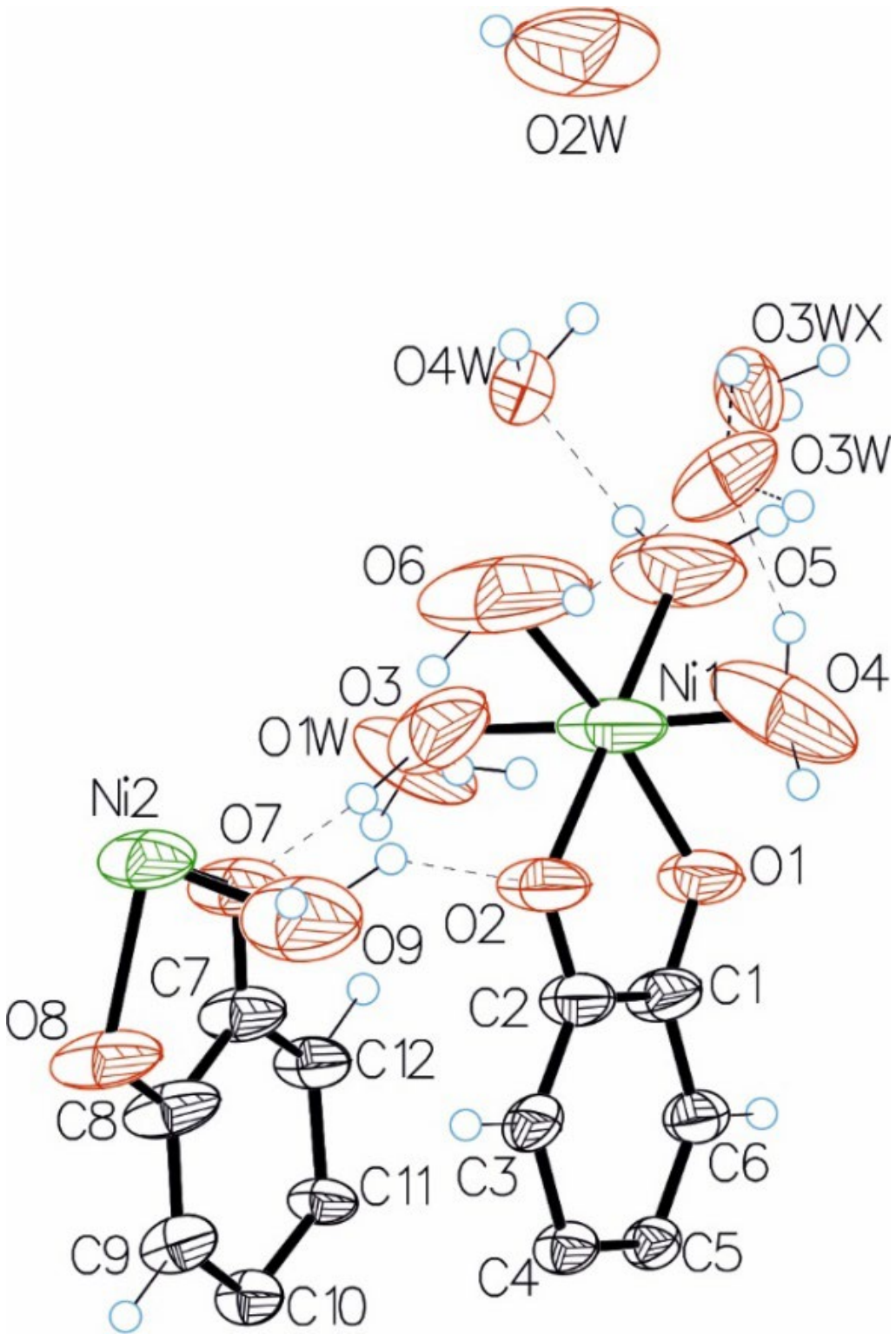

Figure SI11. ORTEP-Drawing (thermal ellipsoids draw at 50% level) showing the atoms in the asymmetric unit of structure Ni-HHTP.

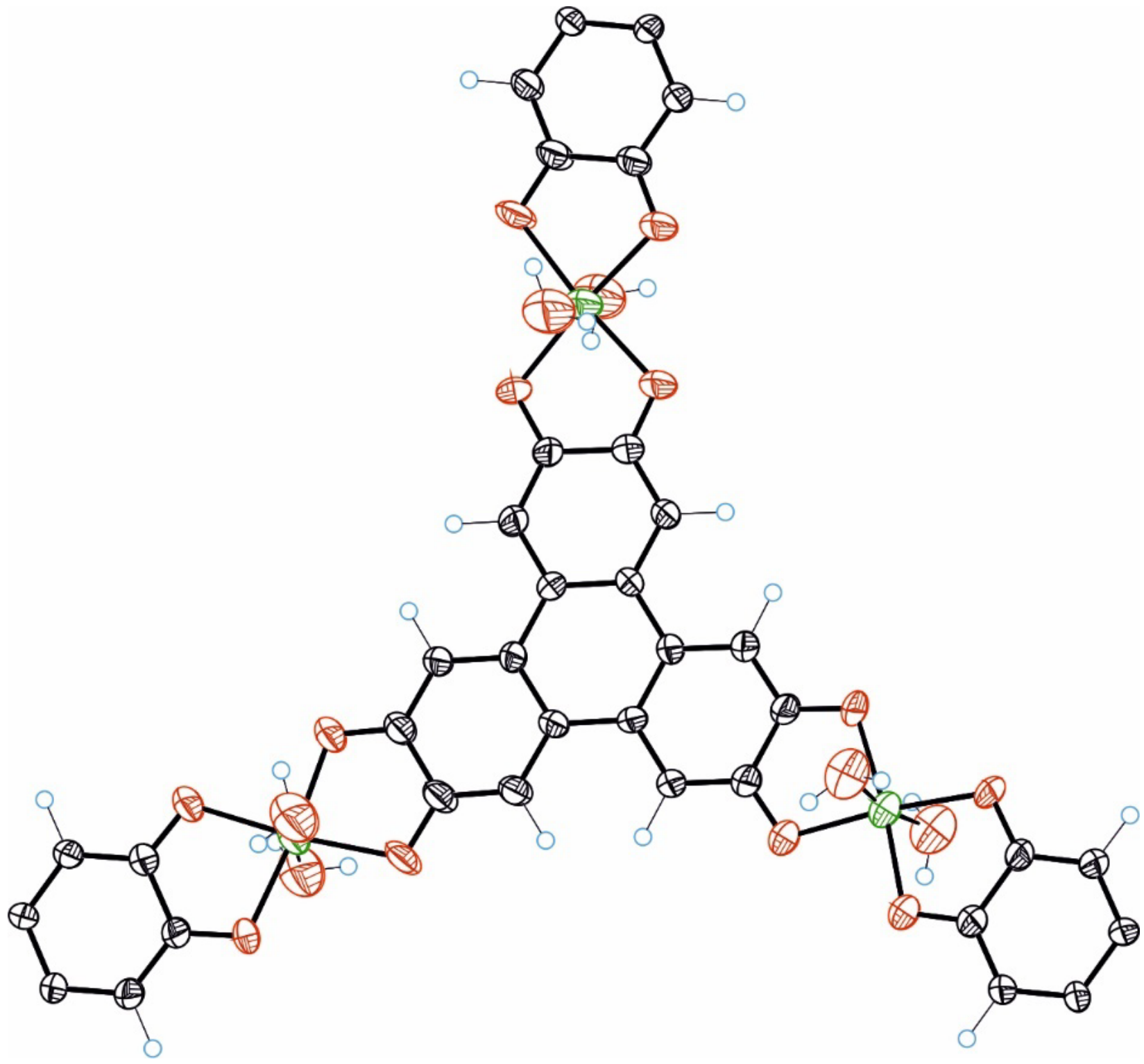

Figure SI12. ORTEP-Drawing (thermal ellipsoids draw at 50% level) showing a fragment of the structure corresponding to Fragment A.

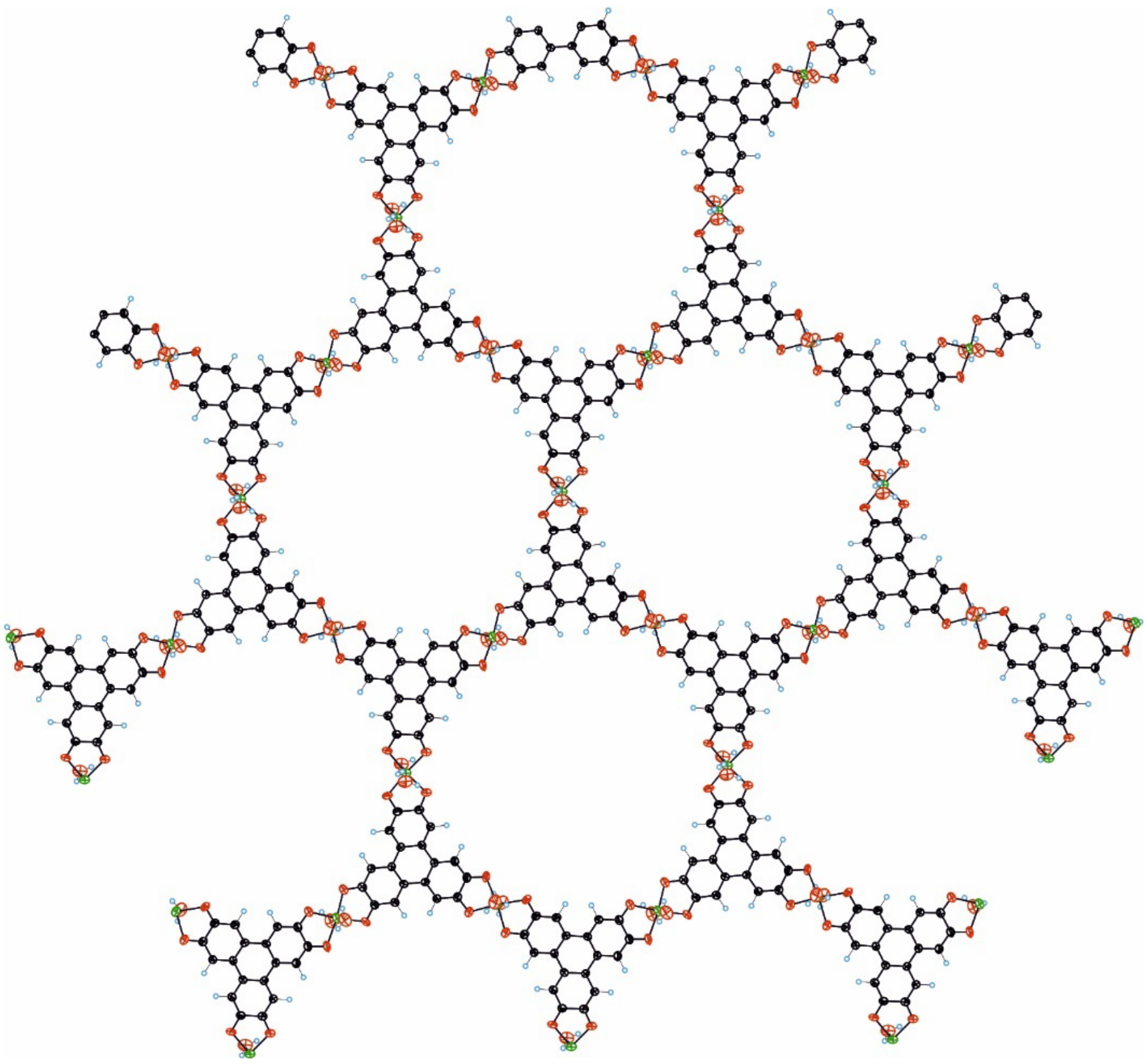

Figure SI13. ORTEP-Drawing (thermal ellipsoids draw at 50% level) showing the two-dimensional polymer formed by Fragment A.

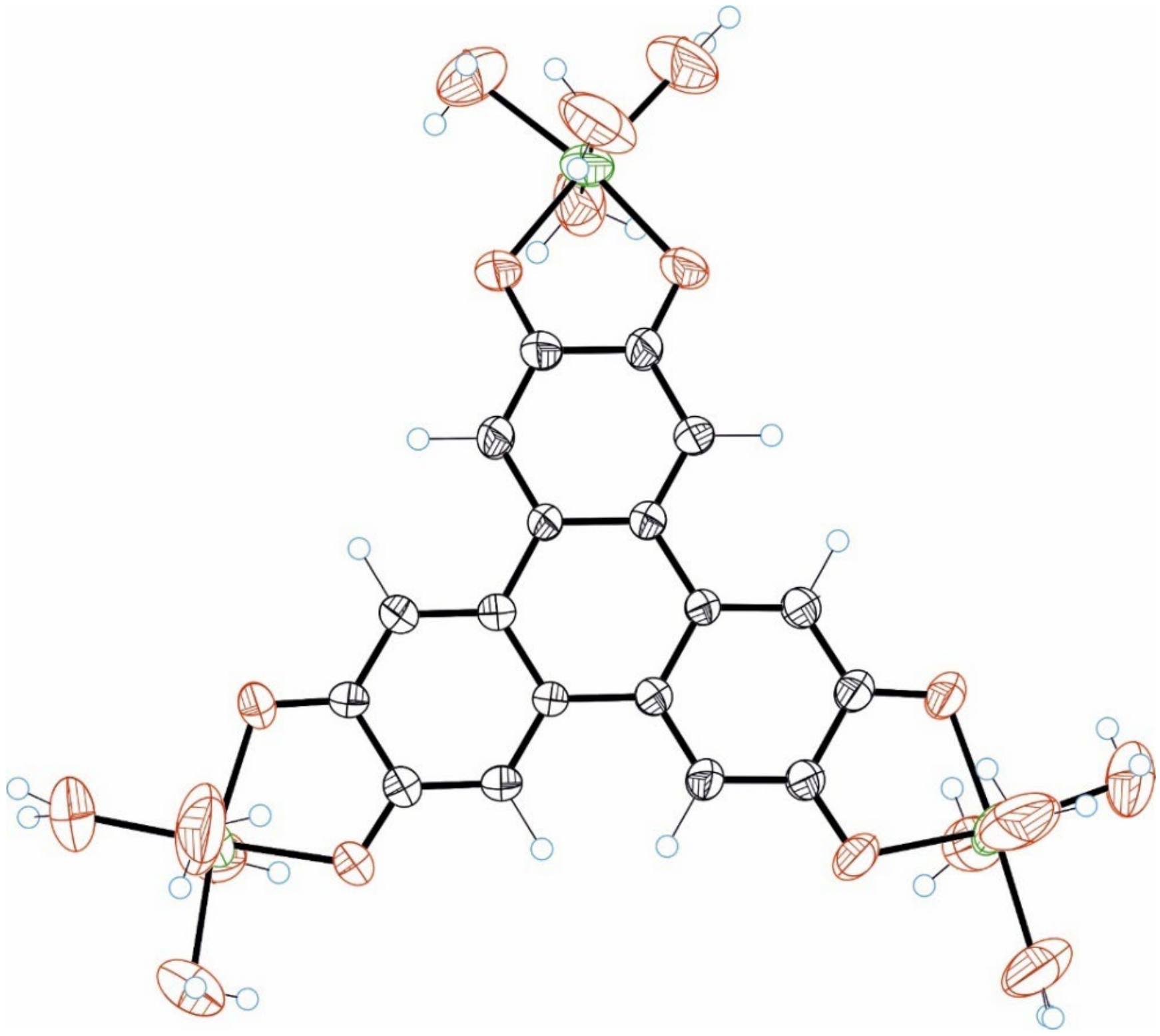

Figure SI14. ORTEP-Drawing (thermal ellipsoids draw at 50% level) showing a fragment of the structure corresponding to Fragment B.

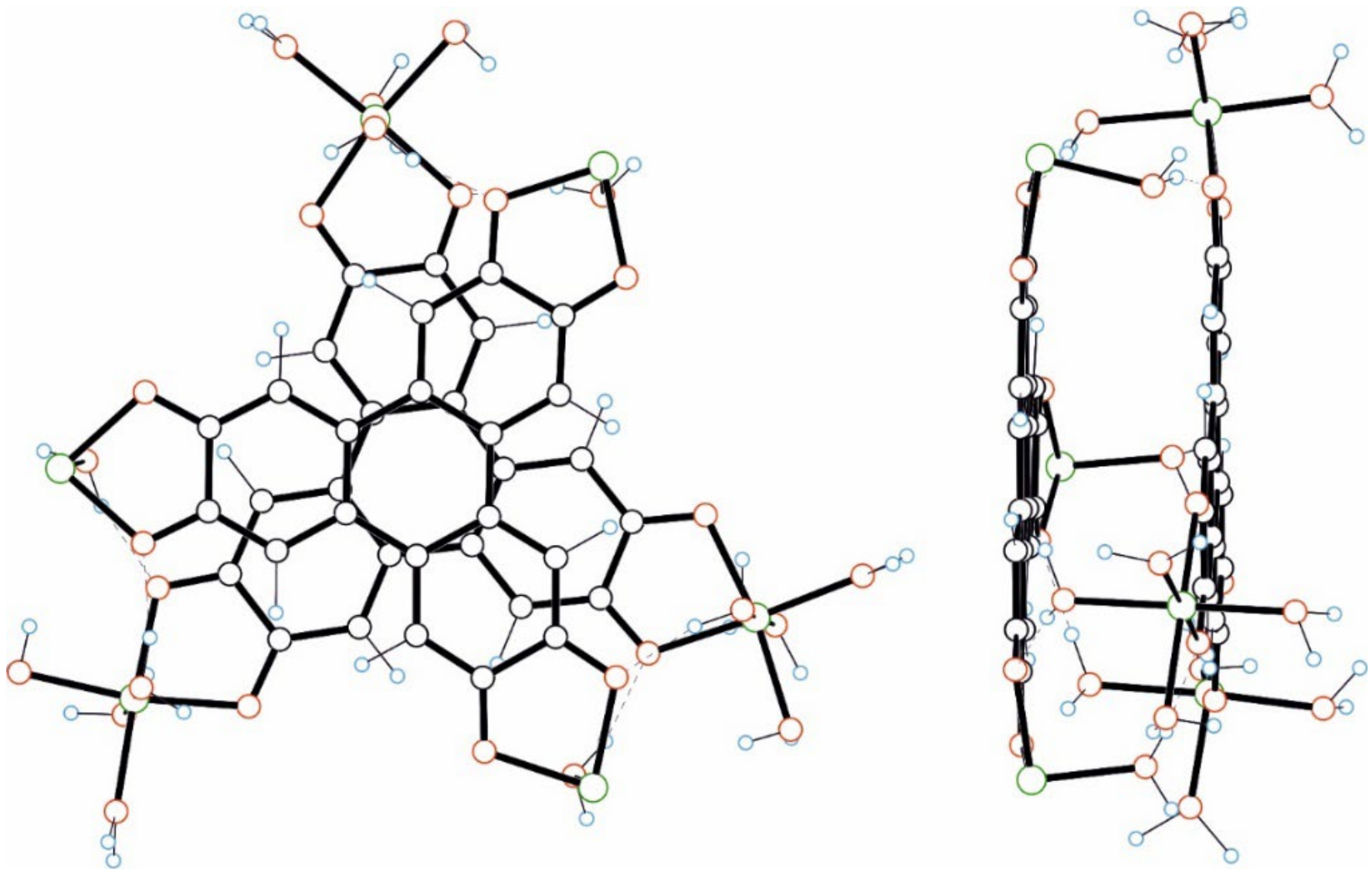

Figure SI15. Drawing showing the interaction between the Fragment A and the Fragment B forming a sandwich like arrangement in a frontal and side view.

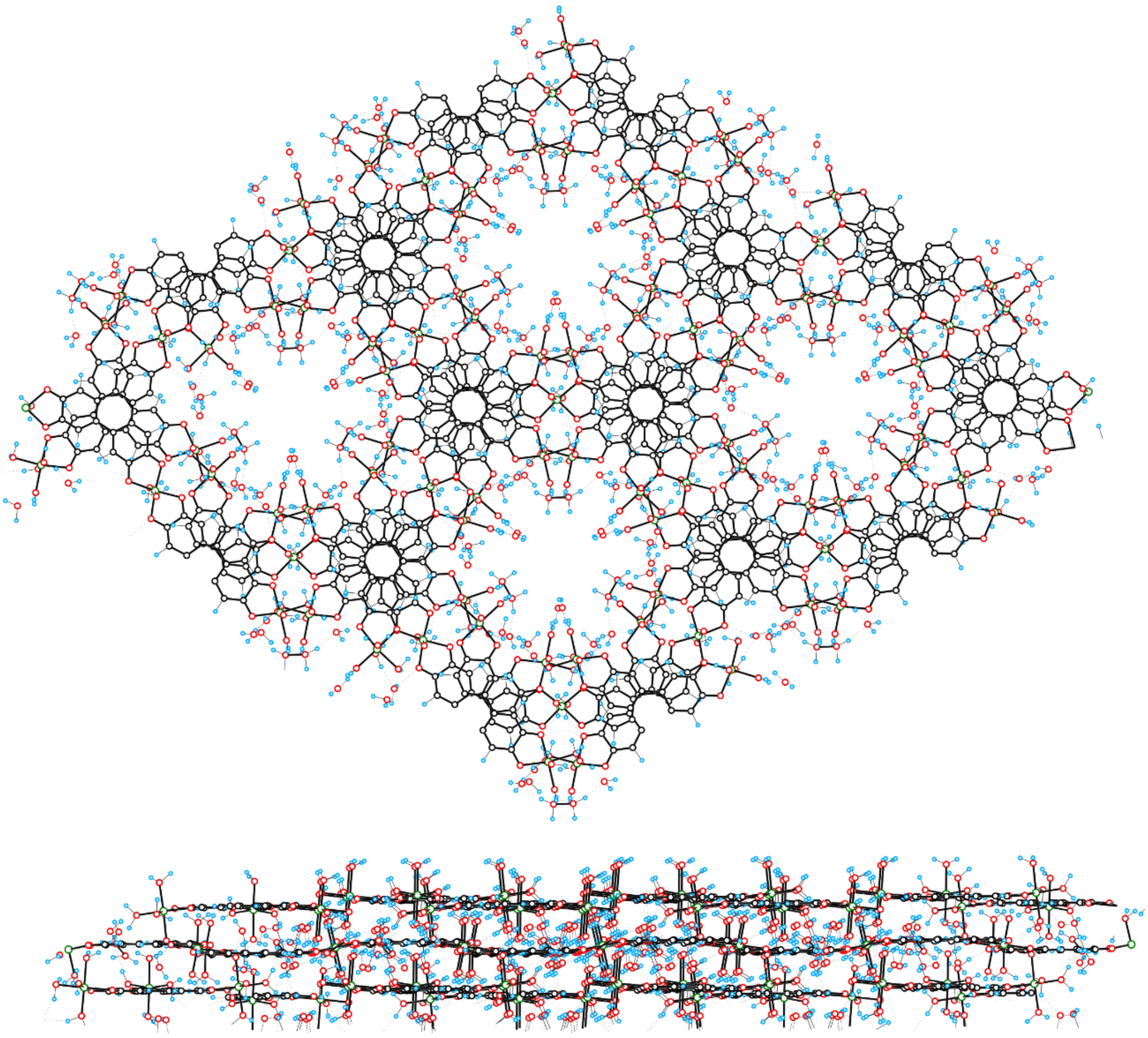

Figure SI16. Drawing showing the whole structure of the crystal.

Table SI2. Data collection parameter overview.

Dataset (grain)	Number of frames	Scan range [°]	Scan width [°]	Exposure time [min]	Total time [min:s]	Total Dose [$e^-/Å^2$]
**07**	488	-62 to +59.75	0.25	1.00	08:19	7.418
**09**	496	-65 to+61.75	0.25	1.00	08:16	7.539
**05**	504	-62 to +63.75	0.25	1.00	08:24	7.661
**Merged**	Crystals	03, 09, 05		Total:	24:59	22.618

Table SI3. Data quality statistics overview for selected single data collections and merged data for crystals 07, 09 and 05 (resolution of 0.80 Å). Point group symmetry: $P2_1$.

Dataset (grain)	Data Fo>4σ	Compl. [%]	Redund.	$<F^2>$	$<F^2/\sigma(F^2)>$	R_{int}	R_{rim}	R_{pim}	CC^*
**07**	1507	99.9	7.3	4.92	2.96	0.240	0.246	0.096	0.996
**09**	1431	100	7.4	3.84	2.77	0.260	0.277	0.106	0.992
**05**	1757	100	7.5	543.98	3.29	0.202	0.204	0.082	0.993
**Merged Crystals 07, 09, 05**	2098	100	22.1	394.71	5.26	0.268	0.270	0.060	0.993

Table SI4. Kinematical refinement parameter for all single data collections and merged data.

Dataset (grain)	R_{int} Olex [%]	R1 [%]	wR2 [%]	Resolution [Å]	Resolution [I/σ(I)]
**07**	25.36	15.56	38.51	0.80	6.7
**09**	28.79	15.71	38.58	0.80	6.5
**05**	21.33	16.14	44.95	0.80	6.3
**merged Crystals 07, 09, 05**	**29.63**	**15.11**	**36.83**	**0.80**	**13.2**

Table SI5. Crystal data and structure refinement for Crystal 07	
Identification code	IF29LT07_P-3c1_JBB80
Empirical formula	$C_{24}H_{33.8}Ni_3O_{20.9}$
Formula weight	832.79
Temperature/K	100
Crystal system	trigonal
Space group	P-3c1
a/Å	21.62(14)
b/Å	21.62(14)
c/Å	12.70(19)
α/°	90
β/°	90
γ/°	120
Volume/Å^3	5137(101)
Z	6
ρ_{calc}g/cm^3	1.615
μ/mm^{-1}	0.000
F(000)	835.0
Crystal size/mm^3	0.0012 × 0.0002 × 0.0001
Radiation	electron (λ = 0.0251)
2Θ range for data collection/°	0.076 to 1.798
Index ranges	-26 ≤ h ≤ 26, -27 ≤ k ≤ 27, -15 ≤ l ≤ 15
Reflections collected	26407
Independent reflections	3505 [R_{int} = 0.2536, R_{sigma} = 0.1491]
Data/restraints/parameters	3505/376/262
Goodness-of-fit on F^2	1.435
Final R indexes [I>=2σ (I)]	R_1 = 0.1556, wR_2 = 0.3460
Final R indexes [all data]	R_1 = 0.2424, wR_2 = 0.3851
Largest diff. peak/hole / e Å^{-3}	0.20/-0.17

Table SI6. Bond Lengths for IF29LT07_P-3c1_JBB80.					
Atom	Atom	Length/Å	Atom	Atom	Length/Å
Ni2	O7	1.969(15)	C10	C11	1.421(12)
Ni2	O7[1]	1.969(15)	C10	C11[3]	1.456(13)
Ni2	O8[1]	2.000(15)	C10	C9	1.372(13)
Ni2	O8	2.000(15)	C11	C12	1.388(13)
Ni2	O9	2.00(3)	C4	C3	1.368(13)
Ni2	O9[1]	2.01(3)	C6	C1	1.407(13)
Ni1	O2	2.001(15)	C9	C8	1.366(13)
Ni1	O1	2.005(15)	C12	C7	1.371(13)
Ni1	O3	2.11(3)	C1	C2	1.455(14)
Ni1	O4	2.03(3)	C1	O1	1.278(12)
Ni1	O5	2.028(17)	C2	O2	1.282(13)
Ni1	O6	2.078(19)	C2	C3	1.382(13)
C5	C4[2]	1.453(13)	C8	C7	1.453(14)
C5	C4	1.439(13)	C8	O8	1.296(12)
C5	C6	1.355(13)	C7	O7	1.269(13)

[1]-X,-X+Y,3/2-Z; [2]1-Y,1+X-Y,+Z; [3]+Y-X,1-X,+Z

Table SI7. Bond Angles for IF29LT07_P-3c1_JBB80.							
**Atom**	**Atom**	**Atom**	**Angle/˚**	**Atom**	**Atom**	**Atom**	**Angle/˚**
O7	Ni2	$O7^{1}$	91.9(4)	C6	C5	C4	120.0(6)
$O7^{1}$	Ni2	O8	174.8(3)	C6	C5	$C4^{2}$	119.9(6)
$O7^{1}$	Ni2	$O8^{1}$	83.0(3)	C11	C10	$C11^{3}$	118.3(7)
O7	Ni2	O8	83.0(3)	C9	C10	$C11^{3}$	121.5(6)
O7	Ni2	$O8^{1}$	174.8(3)	C9	C10	C11	120.0(6)
$O7^{1}$	Ni2	O9	93.7(5)	C10	C11	$C10^{2}$	121.6(7)
$O7^{1}$	Ni2	$O9^{1}$	93.5(5)	C12	C11	C10	118.6(6)
O7	Ni2	$O9^{1}$	93.7(5)	C12	C11	$C10^{2}$	119.7(6)
O7	Ni2	O9	93.5(5)	C5	C4	$C5^{3}$	119.9(6)
O8	Ni2	$O8^{1}$	102.1(4)	C3	C4	C5	118.2(6)
$O8^{1}$	Ni2	O9	87.9(5)	C3	C4	$C5^{3}$	121.9(6)
O8	Ni2	O9	85.6(5)	C5	C6	C1	122.6(7)
O8	Ni2	$O9^{1}$	87.9(5)	C8	C9	C10	122.0(7)
$O8^{1}$	Ni2	$O9^{1}$	85.6(5)	C7	C12	C11	121.7(6)
O9	Ni2	$O9^{1}$	169.7(7)	C6	C1	C2	117.2(7)
O2	Ni1	O1	81.7(3)	O1	C1	C6	124.9(7)
O2	Ni1	O3	91.2(4)	O1	C1	C2	117.9(7)
O2	Ni1	O4	91.7(5)	O2	C2	C1	116.4(6)
O2	Ni1	O5	175.8(6)	O2	C2	C3	124.8(7)
O2	Ni1	O6	89.6(5)	C3	C2	C1	118.8(6)
O1	Ni1	O3	90.6(4)	C9	C8	C7	118.6(6)
O1	Ni1	O4	89.7(6)	O8	C8	C9	123.9(7)
O1	Ni1	O5	95.9(4)	O8	C8	C7	117.5(7)
O1	Ni1	O6	171.0(6)	C12	C7	C8	119.0(6)
O4	Ni1	O3	177.0(4)	O7	C7	C12	123.9(7)
O4	Ni1	O6	92.8(9)	O7	C7	C8	117.1(7)
O5	Ni1	O3	85.3(6)	C7	O7	Ni2	111.8(6)
O5	Ni1	O4	91.8(6)	C2	O2	Ni1	112.3(6)
O5	Ni1	O6	92.7(5)	C4	C3	C2	123.2(7)
O6	Ni1	O3	87.4(8)	C1	O1	Ni1	111.5(6)
C4	C5	$C4^{2}$	120.1(6)	C8	O8	Ni2	109.6(6)

^{1}-X,-X+Y,3/2-Z; ^{2}1-Y,1+X-Y,+Z; ^{3}+Y-X,1-X,+Z

Table SI8. Torsion Angles for IF29LT07_P-3c1_JBB80.									
**A**	**B**	**C**	**D**	**Angle/˚**	**A**	**B**	**C**	**D**	**Angle/˚**
C5[1]	C4	C3	C2	-178.9(7)	C6	C1	C2	O2	-177.8(8)
C5	C4	C3	C2	-0.2(11)	C6	C1	C2	C3	-0.3(12)
C5	C6	C1	C2	0.0(11)	C6	C1	O1	Ni1	173.8(7)
C5	C6	C1	O1	-178.2(9)	C9	C10	C11	C10[2]	178.3(5)
C10[2]	C11	C12	C7	-179.5(8)	C9	C10	C11	C12	-3.8(11)
C10	C11	C12	C7	2.5(11)	C9	C8	C7	C12	1.4(13)
C10	C9	C8	C7	-2.8(12)	C9	C8	C7	O7	-178.2(9)
C10	C9	C8	O8	179.2(9)	C9	C8	O8	Ni2	171.0(7)
C11[1]	C10	C11	C10[2]	3.0(14)	C12	C7	O7	Ni2	-172.3(7)
C11[1]	C10	C11	C12	-179.1(5)	C1	C2	O2	Ni1	3.7(11)
C11	C10	C9	C8	4.0(12)	C1	C2	C3	C4	0.4(12)
C11[1]	C10	C9	C8	179.2(7)	C2	C1	O1	Ni1	-4.4(11)
C11	C12	C7	C8	-1.3(12)	C8	C7	O7	Ni2	7.3(12)
C11	C12	C7	O7	178.2(9)	C7	C8	O8	Ni2	-7.0(11)
C4[2]	C5	C4	C5[1]	-2.1(13)	O2	C2	C3	C4	177.7(9)
C4[2]	C5	C4	C3	179.2(5)	C3	C2	O2	Ni1	-173.8(7)
C4[2]	C5	C6	C1	-179.1(7)	O1	C1	C2	O2	0.5(13)
C4	C5	C6	C1	0.1(11)	O1	C1	C2	C3	178.1(9)
C6	C5	C4	C5[1]	178.7(5)	O8	C8	C7	C12	179.5(9)
C6	C5	C4	C3	0.0(10)	O8	C8	C7	O7	-0.1(14)

[1]+Y-X,1-X,+Z; [2]1-Y,1+X-Y,+Z

Table SI9. Crystal data and structure refinement for Merged data crystals 07, 09 and 05	
Identification code	IF29LT_merge070905_P_3c1_JBB80
Empirical formula	$C_{24}H_{33.8}Ni_3O_{20.9}$
Formula weight	832.79
Temperature/K	100
Crystal system	trigonal
Space group	P-3c1
a/Å	21.62(14)
b/Å	21.62(14)
c/Å	12.70(19)
α/°	90
β/°	90
γ/°	120
Volume/Å^3	5137(101)
Z	6
ρ_{calc}g/cm^3	1.612
μ/mm^{-1}	0.000
F(000)	833.0
Crystal size/mm^3	0.0012 × 0.0002 × 0.0001
Radiation	electron (λ = 0.0251)
2Θ range for data collection/°	0.076 to 1.798
Index ranges	-27 ≤ h ≤ 27, -27 ≤ k ≤ 27, -15 ≤ l ≤ 15
Reflections collected	80323
Independent reflections	3503 [R_{int} = 0.2967, R_{sigma} = 0.0755]
Data/restraints/parameters	3503/374/257
Goodness-of-fit on F^2	1.086
Final R indexes [I>=2σ (I)]	R_1 = 0.1498, wR_2 = 0.3319
Final R indexes [all data]	R_1 = 0.1988, wR_2 = 0.3635
Largest diff. peak/hole / e Å^{-3}	0.18/-0.19

Table SI10. Bond Lengths for IF29LT_merge070905_P_3c1_JBB80.

Atom	Atom	Length/Å	Atom	Atom	Length/Å
Ni2	O7	1.979(14)	C10	C11	1.434(11)
Ni2	O7[1]	1.979(14)	C10	C11[3]	1.444(12)
Ni2	O8	2.007(14)	C10	C9	1.378(12)
Ni2	O8[1]	2.007(14)	C11	C12	1.372(11)
Ni2	O9[1]	2.02(3)	C4	C3	1.376(12)
Ni2	O9	2.02(3)	C6	C1	1.402(12)
Ni1	O2	1.988(14)	C9	C8	1.376(12)
Ni1	O1	2.008(15)	C12	C7	1.379(12)
Ni1	O3	2.09(3)	C1	C2	1.437(13)
Ni1	O4	2.01(3)	C1	O1	1.287(11)
Ni1	O5	2.027(16)	C2	O2	1.278(12)
Ni1	O6	2.063(18)	C2	C3	1.389(12)
C5	C4[2]	1.444(12)	C8	C7	1.459(13)
C5	C4	1.437(12)	C8	O8	1.283(11)
C5	C6	1.369(12)	C7	O7	1.270(11)

[1]-X,-X+Y,3/2-Z; [2]1-Y,1+X-Y,+Z; [3]+Y-X,1-X,+Z

Table SI11. Bond Angles for IF29LT_merge070905_P_3c1_JBB80.							
**Atom**	**Atom**	**Atom**	**Angle/˚**	**Atom**	**Atom**	**Atom**	**Angle/˚**
O7	Ni2	O7[1]	91.6(4)	C6	C5	C4[2]	119.7(5)
O7[1]	Ni2	O8[1]	82.8(2)	C6	C5	C4	119.9(5)
O7	Ni2	O8[1]	174.2(3)	C11	C10	C11[3]	120.1(5)
O7[1]	Ni2	O8	174.2(3)	C9	C10	C11	118.7(5)
O7	Ni2	O8	82.8(2)	C9	C10	C11[3]	121.0(5)
O7[1]	Ni2	O9[1]	93.4(4)	C10	C11	C10[2]	119.9(5)
O7	Ni2	O9[1]	93.3(5)	C12	C11	C10[2]	120.2(5)
O7	Ni2	O9	93.4(4)	C12	C11	C10	119.8(5)
O7[1]	Ni2	O9	93.3(5)	C5	C4	C5[3]	119.5(5)
O8	Ni2	O8[1]	102.8(4)	C3	C4	C5[3]	121.8(5)
O8[1]	Ni2	O9	88.6(4)	C3	C4	C5	118.7(5)
O8	Ni2	O9	85.4(5)	C5	C6	C1	121.6(6)
O8	Ni2	O9[1]	88.6(4)	C8	C9	C10	122.6(5)
O8[1]	Ni2	O9[1]	85.4(5)	C11	C12	C7	121.3(5)
O9[1]	Ni2	O9	170.4(6)	C6	C1	C2	118.7(5)
O2	Ni1	O1	81.4(3)	O1	C1	C6	123.5(6)
O2	Ni1	O3	91.6(4)	O1	C1	C2	117.8(6)
O2	Ni1	O4	91.6(4)	O2	C2	C1	116.5(6)
O2	Ni1	O5	175.9(5)	O2	C2	C3	124.7(6)
O2	Ni1	O6	89.9(4)	C3	C2	C1	118.8(5)
O1	Ni1	O3	91.1(4)	C9	C8	C7	117.9(5)
O1	Ni1	O4	90.3(5)	O8	C8	C9	124.0(6)
O1	Ni1	O5	95.9(4)	O8	C8	C7	118.1(6)
O1	Ni1	O6	171.1(5)	C12	C7	C8	119.5(5)
O4	Ni1	O3	176.6(4)	O7	C7	C12	123.7(6)
O4	Ni1	O5	91.6(5)	O7	C7	C8	116.9(6)
O4	Ni1	O6	91.7(7)	C7	O7	Ni2	111.7(5)
O5	Ni1	O3	85.3(5)	C2	O2	Ni1	112.9(5)
O5	Ni1	O6	92.7(5)	C4	C3	C2	122.4(5)
O6	Ni1	O3	87.3(7)	C1	O1	Ni1	111.1(5)
C4	C5	C4[2]	120.4(5)	C8	O8	Ni2	109.6(5)

[1]-X,-X+Y,3/2-Z; [2]1-Y,1+X-Y,+Z; [3]+Y-X,1-X,+Z

Table SI12. Torsion Angles for IF29LT_merge070905_P_3c1_JBB80.

A	B	C	D	Angle/˚	A	B	C	D	Angle/˚
C5[1]	C4	C3	C2	-179.5(5)	C6	C1	C2	O2	-176.9(6)
C5	C4	C3	C2	-1.2(8)	C6	C1	C2	C3	-0.6(9)
C5	C6	C1	C2	-0.2(9)	C6	C1	O1	Ni1	173.0(5)
C5	C6	C1	O1	-177.8(6)	C9	C10	C11	C10[2]	178.5(4)
C10	C11	C12	C7	3.6(9)	C9	C10	C11	C12	-4.7(8)
C10[2]	C11	C12	C7	-179.6(6)	C9	C8	C7	C12	0.4(10)
C10	C9	C8	C7	-1.6(9)	C9	C8	C7	O7	-178.4(6)
C10	C9	C8	O8	179.5(7)	C9	C8	O8	Ni2	171.5(6)
C11[1]	C10	C11	C10[2]	3.7(11)	C12	C7	O7	Ni2	-171.9(5)
C11[1]	C10	C11	C12	-179.6(4)	C1	C2	O2	Ni1	3.5(9)
C11	C10	C9	C8	3.7(9)	C1	C2	C3	C4	1.4(9)
C11[1]	C10	C9	C8	178.5(6)	C2	C1	O1	Ni1	-4.6(9)
C11	C12	C7	C8	-1.5(9)	C8	C7	O7	Ni2	6.8(8)
C11	C12	C7	O7	177.2(6)	C7	C8	O8	Ni2	-7.5(9)
C4[2]	C5	C4	C5[1]	-2.3(9)	O2	C2	C3	C4	177.3(6)
C4[2]	C5	C4	C3	179.4(4)	C3	C2	O2	Ni1	-172.5(5)
C4	C5	C6	C1	0.4(8)	O1	C1	C2	O2	0.8(10)
C4[2]	C5	C6	C1	-178.7(5)	O1	C1	C2	C3	177.1(6)
C6	C5	C4	C5[1]	178.6(4)	O8	C8	C7	C12	179.4(7)
C6	C5	C4	C3	0.3(7)	O8	C8	C7	O7	0.6(11)

[1]+Y-X,1-X,+Z; [2]1-Y,1+X-Y,+Z

Measurements

Figure SI17 presents the intermolecular distances between centroids of HHTP-based units within the crystal structure.

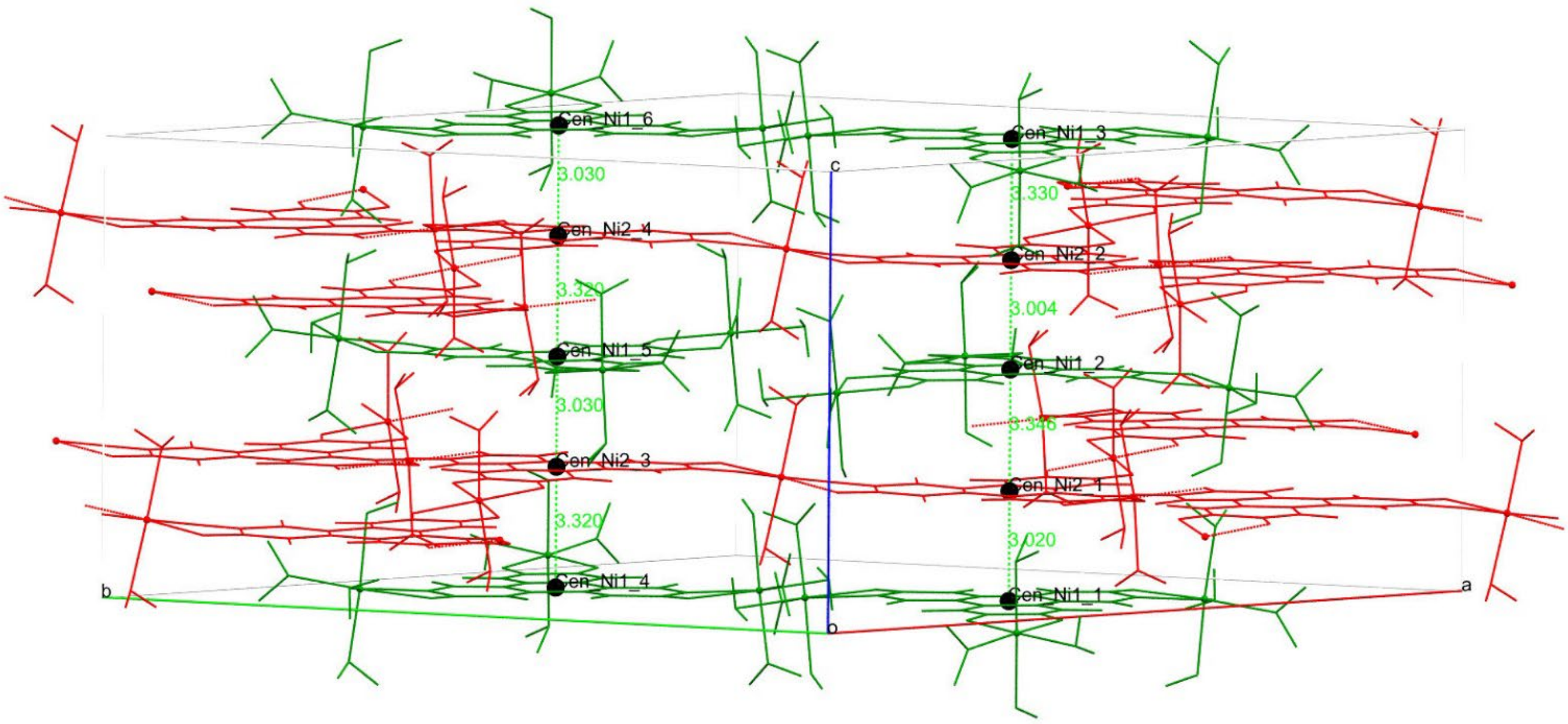

Figure SI17. Distances between centroids of HHTP-based units within the crystal structure shown in different orientations.

8.- Computational Studies

The geometries and energies of all systems included in this study were fully optimized at the RI-UBP86-D4/def2-TZVP level of theory[14,15,16,17] without symmetry constrains. The calculations have been performed by using the program TURBOMOLE version 7.7.[18] The binding energies were computed as the difference between the energy of the assembly minus the sum of the energies of the monomers. The MEP surfaces were computed at the same level of theory and represented using the 0.001 a.u. isosurface.

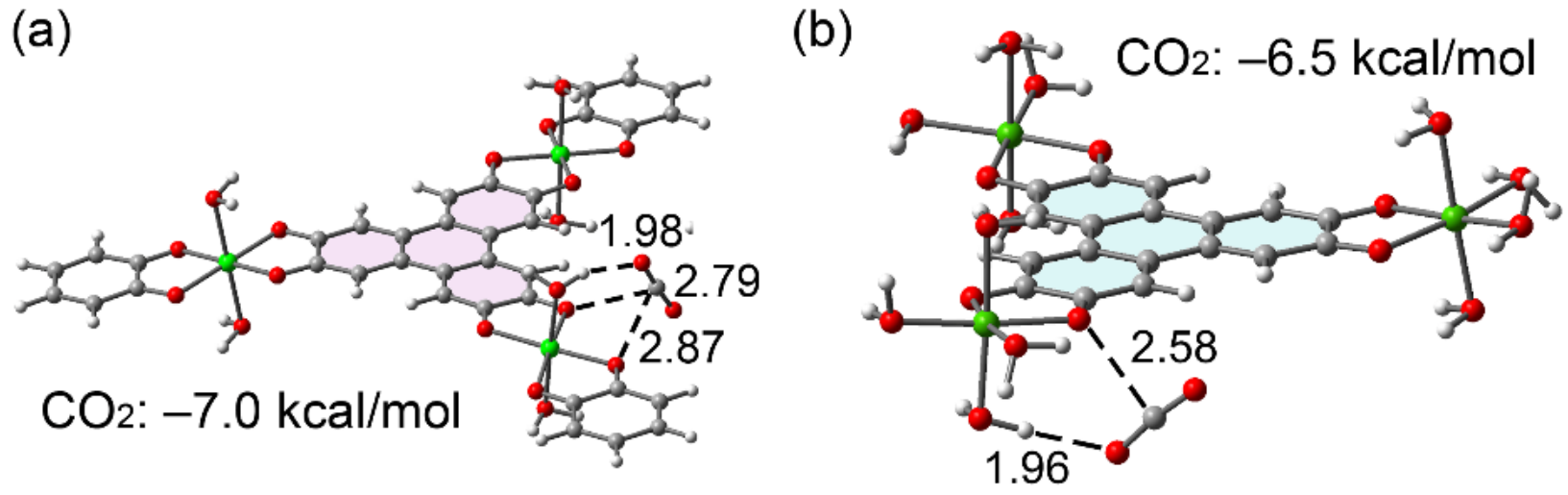

Figure SI18: (a) Optimized geometry of and interaction energy of Fragment A with CO_2. (d) Optimized geometry of and interaction energy of Fragment B with CO_2. Distances in Å

[14] A. D. Becke, "Density-Functional Exchange-Energy Approximation with Correct Asymptotic Behaviour", *Phys. Rev. A* **1988**, *38*, 3098–3100.

[15] J. P. Perdew, "Density-Functional Approximation for the Correlation Energy of the Inhomogeneous Electron Gas", *Phys. Rev. B* **1986**, *33*, 8822–8824.

[16] F. Weigend, "Accurate Coulomb-Fitting Basis Sets for H to Rn", *Phys. Chem. Chem. Phys.* **2006**, *8*, 1057–1065.

[17] E. Caldeweyher; S. Ehlert; A. Hansen; H. Neugebauer; S. Spicher; C. Bannwarth; S. A. Grimme, "Generally Applicable Atomic-Charge Dependent London Dispersion Correction", *J. Chem. Phys.* **2019**, *150* (15), 154122.

[18] R. Ahlrichs; M. Bär; M. Häser; H. Horn; C. Kölmel, "Electronic Structure Calculations on Workstation Computers: The Program System Turbomole", *Chem. Phys. Let.* **1989**, *162*, 165–169.

9. Cartesian coordinates

MOF "A"

Energy = -7271.454230238

Ni	-5.5704971	9.4540681	9.5797864
C	-2.8503913	12.5376659	9.8497244
H	-3.4087036	13.4712761	9.8372639
C	-3.6147293	11.3570987	9.8339561
C	-2.9143625	10.0680421	9.8505065
O	-3.6302702	8.9898582	9.8656258
O	-4.9088566	11.3430021	9.8294270
O	-5.2836202	9.5652222	7.4449764
H	-6.1287206	9.9822983	7.1893993
H	-5.3812813	8.6199265	7.2207889
C	-1.3390768	15.0031649	9.8819438
H	-2.4245713	15.0768800	9.8839382
C	0.8315081	16.1821720	9.8663145
C	-0.6349306	16.2202636	9.8722831
O	-1.2111252	17.3793024	9.8891706
O	1.4668735	17.3098991	9.8747779
O	-5.8519252	9.3487743	11.7141069
H	-5.7441205	10.2960995	11.9257738
H	-5.0052082	8.9303251	11.9624088
C	-10.3409904	7.6589615	9.0583336
C	-9.6608602	6.4080517	9.0823622
C	-9.6478521	8.8503783	9.1260610
H	-10.1571274	9.8146773	9.1112802
C	-8.2853429	6.3448551	9.1746120
H	-7.7527162	5.3933627	9.1969021
C	-8.2299844	8.8387100	9.2257899
C	-7.5259052	7.5441987	9.2513114
O	-6.2359525	7.5639119	9.3224693
O	-7.5135566	9.9130548	9.2755369
C	0.9933238	23.8467311	9.0609424
C	-0.4301921	23.8817480	9.0718749
C	1.6788674	22.6513515	9.1350025
H	2.7686611	22.6111376	9.1309635
C	-1.1723362	22.7213366	9.1559132
H	-2.2627144	22.7350029	9.1690327
C	0.9600869	21.4287016	9.2283637
C	-0.5130257	21.4647695	9.2383317
Ni	5.4100144	9.1741781	9.5755252
C	0.6971454	11.2088098	9.8660392
C	1.4556159	12.4461225	9.8706715
C	1.3819294	9.9911980	9.8526732
H	0.8527348	9.0406263	9.8414515
C	2.8512778	12.3886382	9.8674517
H	3.4570973	13.2920484	9.8698785
C	2.7862010	9.9192108	9.8361979

C	3.5529033	11.1700274	9.8465855
O	4.8445543	11.0886985	9.8571888
O	3.4450269	8.8049940	9.8347964
O	5.1555597	9.3484617	7.4411446
H	5.2087191	8.4040138	7.1990119
H	6.0230085	9.7278777	7.2025229
C	0.7595150	13.7279407	9.8752606
C	-0.6909638	13.7657223	9.8749768
C	1.4713896	14.9299675	9.8757532
H	2.5592281	14.9467551	9.8705628
C	-1.4535064	12.5222938	9.8654126
C	-0.7608605	11.2471576	9.8691741
C	-1.5082595	10.0669573	9.8710919
H	-1.0284521	9.0907451	9.8775461
O	5.6580198	9.0038866	11.7093924
H	4.7858817	8.6273416	11.9361215
H	5.5988705	9.9508588	11.9406410
C	9.3402520	5.9288252	9.0619011
C	10.0862302	7.1417826	9.0772380
C	7.9620980	5.9370485	9.1325446
H	7.3792841	5.0152634	9.1244492
C	9.4563931	8.3667042	9.1628958
H	10.0165024	9.3022550	9.1784177
C	7.2665877	7.1731351	9.2266923
C	8.0384294	8.4285907	9.2418527
O	7.3789945	9.5380866	9.3067184
O	5.9784631	7.2592681	9.2798852
Ni	0.1630686	18.8226645	9.5957805
O	0.1860375	19.1516824	11.7260728
H	0.9506990	18.5899746	11.9576919
H	-0.6016895	18.6267073	11.9664665
O	0.1401977	18.4818610	7.4653902
H	0.9272467	19.0009421	7.2113113
H	-0.6264771	19.0350471	7.2211242
O	-1.1408600	20.3372055	9.3040577
O	1.5332587	20.2718884	9.2868759
H	1.5461071	24.7848686	8.9952160
H	-0.9374376	24.8459568	9.0146137
H	-11.4294292	7.6690924	8.9869688
H	-10.2429745	5.4870144	9.0284157
H	11.1747157	7.0952006	9.0220519
H	9.8735441	4.9795001	8.9951393

MOF “B” +CO2

Energy = -6773.861502964

Ni	-2.7009000	6.7698000	6.7945000
C	0.1049000	11.0412000	6.5634000
C	-1.1923000	11.6628000	6.5481000
C	0.2086000	9.6894000	6.5761000

H	1.1775529	9.1891168	6.5961703
C	-0.9145000	8.8412000	6.5735000
C	-2.2247000	9.4741000	6.5557000
O	-3.2365000	8.6877000	6.5964000
C	-2.2982000	10.8559000	6.5469000
H	-3.3045734	11.2773047	6.5453889
O	-0.8670000	7.5643000	6.6180000
O	-2.6074000	6.9651000	8.8913000
H	-3.1923812	7.7449668	8.9751665
H	-1.7114046	7.3650479	8.8309312
O	-2.7565000	6.4821000	4.7828000
H	-1.7887577	6.6090127	4.6649364
H	-3.1063049	7.3817350	4.5946500
O	-2.0734000	4.8681000	7.1120000
H	-2.2555106	4.6960220	8.0548443
H	-1.1011680	4.9422518	7.0507367
O	-4.7013000	6.2518000	7.0231000
H	-5.0229978	5.7263015	6.2684031
H	-5.0397258	7.1595495	6.8733326
C	1.1956000	13.2937000	6.5634000
C	1.3058000	11.8594000	6.5481000
C	2.3144000	14.0595000	6.5761000
H	2.2610046	15.1485528	6.5950383
C	3.6105000	13.5109000	6.5735000
C	3.7176000	12.0598000	6.5557000
O	4.9045000	11.5767000	6.5964000
C	2.5576000	11.3052000	6.5469000
H	2.6919492	10.2222008	6.5667528
O	4.6926000	14.1905000	6.6180000
C	-1.3004000	13.1120000	6.5634000
C	-0.1135000	13.9246000	6.5481000
C	-2.5231000	13.6981000	6.5761000
H	-3.4401525	13.1082040	6.5970320
C	-2.6960000	15.0949000	6.5735000
C	-1.4929000	15.9131000	6.5557000
O	-1.6680000	17.1825000	6.5964000
C	-0.2594000	15.2858000	6.5469000
H	0.6081070	15.9477792	6.5457608
O	-3.8257000	15.6921000	6.6180000
Ni	6.2976000	12.9995000	6.7945000
O	6.0817000	12.9829000	8.8913000
H	5.6971628	12.0885842	8.9903095
H	5.2882006	13.5597778	8.8231277
O	6.5746000	13.0952000	4.7828000
H	5.9259425	13.8270213	4.6830676
H	6.0721985	12.3153635	4.4513266
O	7.6308000	14.4938000	7.1120000
H	7.8572254	14.4438016	8.0596588
H	7.0839497	15.2983626	7.0221706
O	7.7464000	11.5262000	7.0231000
H	8.3448315	11.4897845	6.2552094

H	7.1506155	10.7550644	6.9315894
Ni	-3.5967000	17.6776000	6.7945000
O	-3.4743000	17.4990000	8.8913000
H	-2.5077268	17.6206074	8.9819467
H	-3.5704728	16.5228868	8.8226047
O	-3.8181000	17.8697000	4.7828000
H	-4.1890848	16.9660526	4.6688871
H	-2.8633629	17.7243122	4.5976241
O	-5.5574000	18.0850000	7.1120000
H	-5.6126525	18.3303567	8.0546533
H	-5.9812762	17.2067542	7.0528986
O	-3.0452000	19.6690000	7.0231000
H	-3.3399278	20.2104038	6.2686072
H	-2.0911813	19.5032668	6.8696894
C	5.0845594	9.7995300	4.7305738
O	4.9722456	8.8762694	5.4393475
O	5.2393283	10.6239749	3.9030916

MOF “B” +CO2 (Local Minimum)

Energy = -6773.857375736

Ni	-2.7009000	6.7698000	6.7945000
C	0.1049000	11.0412000	6.5634000
C	-1.1923000	11.6628000	6.5481000
C	0.2086000	9.6894000	6.5761000
H	1.1776971	9.1904562	6.6005925
C	-0.9145000	8.8412000	6.5735000
C	-2.2247000	9.4741000	6.5557000
O	-3.2365000	8.6877000	6.5964000
C	-2.2982000	10.8559000	6.5469000
H	-3.3046420	11.2773231	6.5460684
O	-0.8670000	7.5643000	6.6180000
O	-2.6074000	6.9651000	8.8913000
H	-3.2001067	7.7386246	8.9785911
H	-1.7158626	7.3742662	8.8240018
O	-2.7565000	6.4821000	4.7828000
H	-1.7883975	6.6152439	4.6717729
H	-3.1096184	7.3807471	4.5957725
O	-2.0734000	4.8681000	7.1120000
H	-2.2487835	4.7020783	8.0572470
H	-1.1017427	4.9457916	7.0444449
O	-4.7013000	6.2518000	7.0231000
H	-5.0190558	5.7237272	6.2685286
H	-5.0375108	7.1594462	6.8673674
C	1.1956000	13.2937000	6.5634000
C	1.3058000	11.8594000	6.5481000
C	2.3144000	14.0595000	6.5761000
H	2.2606488	15.1485316	6.5965423
C	3.6105000	13.5109000	6.5735000
C	3.7176000	12.0598000	6.5557000

O	4.9045000	11.5767000	6.5964000
C	2.5576000	11.3052000	6.5469000
H	2.6954501	10.2238385	6.5543185
O	4.6926000	14.1905000	6.6180000
C	-1.3004000	13.1120000	6.5634000
C	-0.1135000	13.9246000	6.5481000
C	-2.5231000	13.6981000	6.5761000
H	-3.4400369	13.1079835	6.5969024
C	-2.6960000	15.0949000	6.5735000
C	-1.4929000	15.9131000	6.5557000
O	-1.6680000	17.1825000	6.5964000
C	-0.2594000	15.2858000	6.5469000
H	0.6080257	15.9478948	6.5462127
O	-3.8257000	15.6921000	6.6180000
Ni	6.2976000	12.9995000	6.7945000
O	6.0817000	12.9829000	8.8913000
H	5.7245035	12.0764125	8.9783523
H	5.2732208	13.5383019	8.8288234
O	6.5746000	13.0952000	4.7828000
H	5.9562380	13.8518584	4.6705657
H	5.9908813	12.3247016	4.6021452
O	7.6308000	14.4938000	7.1120000
H	7.8570929	14.4288557	8.0589320
H	7.0781205	15.2960624	7.0379500
O	7.7464000	11.5262000	7.0231000
H	8.3910131	11.5243977	6.2930974
H	7.1780892	10.7407990	6.8717709
Ni	-3.5967000	17.6776000	6.7945000
O	-3.4743000	17.4990000	8.8913000
H	-2.5085988	17.6274743	8.9815821
H	-3.5635917	16.5223345	8.8223462
O	-3.8181000	17.8697000	4.7828000
H	-4.1872440	16.9649728	4.6710209
H	-2.8627043	17.7248097	4.5999912
O	-5.5574000	18.0850000	7.1120000
H	-5.6111537	18.3220494	8.0569180
H	-5.9803653	17.2067093	7.0466896
O	-3.0452000	19.6690000	7.0231000
H	-3.3432641	20.2101543	6.2697810
H	-2.0919367	19.5026937	6.8655697
O	3.8897110	7.7026300	6.2514079
C	4.9515863	8.1801549	6.3648869
O	6.0342995	8.6188256	6.4778004

MOF “A” + H2O

Energy = -7347.949692503

Ni	-5.6750189	9.5136851	9.5590890
C	-2.9240129	12.5839400	9.7221511
H	-3.4729987	13.5228325	9.6987464

C	-3.6995750	11.4113405	9.7286304
C	-3.0096057	10.1172083	9.7601871
O	-3.7315919	9.0449687	9.7983314
O	-4.9938074	11.4068734	9.7340057
O	-5.4547014	9.5496711	7.4176364
H	-6.3109777	9.9500094	7.1722642
H	-5.5452747	8.5981994	7.2188097
C	-1.3898107	15.0356726	9.7486034
H	-2.4746276	15.1196432	9.7555498
C	0.7928481	16.1956735	9.7423226
C	-0.6735115	16.2467498	9.7466913
O	-1.2393789	17.4096592	9.7739640
O	1.4373173	17.3177880	9.7666214
O	-5.9170354	9.5061507	11.7016905
H	-5.7855720	10.4599355	11.8665160
H	-5.0834577	9.0776343	11.9741234
C	-10.4648611	7.7217537	9.2895204
C	-9.7884592	6.4701768	9.3552373
C	-9.7661137	8.9116206	9.2693117
H	-10.2728683	9.8761626	9.2214746
C	-8.4109032	6.4047415	9.4008274
H	-7.8805790	5.4531357	9.4531030
C	-8.3457971	8.8980319	9.3197703
C	-7.6455739	7.6027236	9.3872787
O	-6.3538602	7.6190334	9.4106637
O	-7.6245216	9.9701126	9.2890516
C	1.0178368	23.8865096	9.2473678
C	-0.4054669	23.9315333	9.2504418
C	1.6945316	22.6839049	9.2627019
H	2.7839917	22.6361513	9.2632923
C	-1.1563529	22.7739547	9.2691437
H	-2.2466873	22.7946745	9.2747686
C	0.9665734	21.4633604	9.2881519
C	-0.5063384	21.5101292	9.2919897
Ni	5.3376742	9.1443495	9.5186945
C	0.6120047	11.2245209	9.7453488
C	1.3811458	12.4544678	9.7251336
C	1.2854983	9.9990021	9.7558740
H	0.7426945	9.0562591	9.7771150
C	2.7748266	12.3808338	9.7033224
H	3.3913810	13.2764996	9.6746418
C	2.6842762	9.9126401	9.7380650
C	3.4650357	11.1527788	9.6980484
O	4.7543425	11.0654795	9.6817391
O	3.3375857	8.7881779	9.7761658
O	4.9945681	9.1041455	7.4427748
H	4.5442706	8.2001545	7.3598908
H	5.8558688	9.0417865	6.9946000
C	0.6971172	13.7426765	9.7318577
C	-0.7529532	13.7926833	9.7366781
C	1.4204684	14.9377726	9.7391696

H	2.5084165	14.9433968	9.7374469
C	-1.5270386	12.5564499	9.7351046
C	-0.8447210	11.2764516	9.7520076
C	-1.6020766	10.1029941	9.7704251
H	-1.1324061	9.1219881	9.7850618
O	5.5434198	9.1765879	11.6832032
H	4.6660775	8.8307441	11.9347287
H	5.4903543	10.1419152	11.8210207
C	9.2386110	5.8103746	9.6140298
C	9.9958602	7.0101325	9.4969544
C	7.8577354	5.8348007	9.6313416
H	7.2695957	4.9210351	9.7250511
C	9.3752593	8.2373870	9.3956753
H	9.9413755	9.1649243	9.3059261
C	7.1746679	7.0735321	9.5343207
C	7.9544148	8.3157613	9.4095244
O	7.3097778	9.4284604	9.3029765
O	5.8830532	7.1806859	9.5352065
Ni	0.1475039	18.8510970	9.5337843
O	0.1705171	19.1074369	11.6757568
H	0.9260696	18.5259216	11.8874417
H	-0.6257397	18.5908878	11.9052275
O	0.1362171	18.5947143	7.3942553
H	0.9366326	19.1051856	7.1654209
H	-0.6155642	19.1741965	7.1652067
O	-1.1429385	20.3854698	9.2974131
O	1.5307606	20.3005352	9.2891886
H	1.5775213	24.8228103	9.2351772
H	-0.9051472	24.9013241	9.2403035
H	-11.5551282	7.7335101	9.2567768
H	-10.3754531	5.5508693	9.3713210
H	11.0849486	6.9505785	9.4899848
H	9.7636137	4.8573961	9.6929008
O	3.7388164	6.7904743	7.8321469
H	3.1847395	7.2793119	8.4819878
H	4.4827194	6.4902691	8.4040807

MOF “B” + N2

Energy = -6694.744150469

Ni	-2.7009000	6.7698000	6.7945000
C	0.1049000	11.0412000	6.5634000
C	-1.1923000	11.6628000	6.5481000
C	0.2086000	9.6894000	6.5761000
H	1.1776971	9.1904562	6.6005925
C	-0.9145000	8.8412000	6.5735000
C	-2.2247000	9.4741000	6.5557000
O	-3.2365000	8.6877000	6.5964000
C	-2.2982000	10.8559000	6.5469000
H	-3.3045284	11.2775062	6.5460402

O	-0.8670000	7.5643000	6.6180000
O	-2.6074000	6.9651000	8.8913000
H	-3.1922634	7.7450276	8.9756096
H	-1.7114046	7.3650479	8.8309312
O	-2.7565000	6.4821000	4.7828000
H	-1.7888407	6.6154203	4.6695371
H	-3.1106531	7.3799877	4.5943971
O	-2.0734000	4.8681000	7.1120000
H	-2.2532170	4.6979428	8.0556604
H	-1.1014210	4.9439029	7.0486902
O	-4.7013000	6.2518000	7.0231000
H	-5.0204876	5.7252817	6.2680513
H	-5.0384724	7.1595642	6.8704005
C	1.1956000	13.2937000	6.5634000
C	1.3058000	11.8594000	6.5481000
C	2.3144000	14.0595000	6.5761000
H	2.2605785	15.1485505	6.5961160
C	3.6105000	13.5109000	6.5735000
C	3.7176000	12.0598000	6.5557000
O	4.9045000	11.5767000	6.5964000
C	2.5576000	11.3052000	6.5469000
H	2.6954501	10.2238385	6.5543185
O	4.6926000	14.1905000	6.6180000
C	-1.3004000	13.1120000	6.5634000
C	-0.1135000	13.9246000	6.5481000
C	-2.5231000	13.6981000	6.5761000
H	-3.4401760	13.1081925	6.5965587
C	-2.6960000	15.0949000	6.5735000
C	-1.4929000	15.9131000	6.5557000
O	-1.6680000	17.1825000	6.5964000
C	-0.2594000	15.2858000	6.5469000
H	0.6082309	15.9475908	6.5457504
O	-3.8257000	15.6921000	6.6180000
Ni	6.2976000	12.9995000	6.7945000
O	6.0817000	12.9829000	8.8913000
H	5.7181072	12.0799682	8.9886606
H	5.2761756	13.5424611	8.8259824
O	6.5746000	13.0952000	4.7828000
H	5.9787301	13.8694269	4.6707906
H	5.9682203	12.3426567	4.5998708
O	7.6308000	14.4938000	7.1120000
H	7.8778491	14.4153342	8.0526609
H	7.0724871	15.2941784	7.0628495
O	7.7464000	11.5262000	7.0231000
H	8.3583943	11.5130842	6.2651124
H	7.1250412	10.7836375	6.8701453
Ni	-3.5967000	17.6776000	6.7945000
O	-3.4743000	17.4990000	8.8913000
H	-2.5087553	17.6273958	8.9829229
H	-3.5637843	16.5223478	8.8225074
O	-3.8181000	17.8697000	4.7828000

H	-4.1884965	16.9655875	4.6705431
H	-2.8630094	17.7241258	4.5992554
O	-5.5574000	18.0850000	7.1120000
H	-5.6113984	18.3226896	8.0567388
H	-5.9804263	17.2067126	7.0471259
O	-3.0452000	19.6690000	7.0231000
H	-3.3433749	20.2103101	6.2699316
H	-2.0917969	19.5034922	6.8657460
N	3.9506582	7.7305732	5.1927289
N	3.8873141	7.7015311	6.2930425

MOF “B” + CH4

Energy = -6625.700500626

Ni	-2.7009000	6.7698000	6.7945000
C	0.1049000	11.0412000	6.5634000
C	-1.1923000	11.6628000	6.5481000
C	0.2086000	9.6894000	6.5761000
H	1.1778969	9.1898530	6.5972843
C	-0.9145000	8.8412000	6.5735000
C	-2.2247000	9.4741000	6.5557000
O	-3.2365000	8.6877000	6.5964000
C	-2.2982000	10.8559000	6.5469000
H	-3.3046143	11.2773455	6.5449812
O	-0.8670000	7.5643000	6.6180000
O	-2.6074000	6.9651000	8.8913000
H	-3.2096002	7.7306513	8.9824359
H	-1.7208957	7.3850044	8.8259496
O	-2.7565000	6.4821000	4.7828000
H	-1.7887119	6.6232014	4.6771362
H	-3.1152455	7.3779916	4.5934105
O	-2.0734000	4.8681000	7.1120000
H	-2.2390621	4.7069256	8.0598795
H	-1.1023808	4.9428993	7.0341771
O	-4.7013000	6.2518000	7.0231000
H	-5.0158857	5.7183852	6.2709854
H	-5.0388464	7.1578504	6.8613976
C	1.1956000	13.2937000	6.5634000
C	1.3058000	11.8594000	6.5481000
C	2.3144000	14.0595000	6.5761000
H	2.2606432	15.1485033	6.5977093
C	3.6105000	13.5109000	6.5735000
C	3.7176000	12.0598000	6.5557000
O	4.9045000	11.5767000	6.5964000
C	2.5576000	11.3052000	6.5469000
H	2.6967077	10.2226143	6.5469937
O	4.6926000	14.1905000	6.6180000
C	-1.3004000	13.1120000	6.5634000
C	-0.1135000	13.9246000	6.5481000
C	-2.5231000	13.6981000	6.5761000

H	-3.4400318	13.1080245	6.5981316
C	-2.6960000	15.0949000	6.5735000
C	-1.4929000	15.9131000	6.5557000
O	-1.6680000	17.1825000	6.5964000
C	-0.2594000	15.2858000	6.5469000
H	0.6081146	15.9477498	6.5448051
O	-3.8257000	15.6921000	6.6180000
Ni	6.2976000	12.9995000	6.7945000
O	6.0817000	12.9829000	8.8913000
H	5.7598801	12.0638666	8.9825939
H	5.2543131	13.5096530	8.8344830
O	6.5746000	13.0952000	4.7828000
H	5.9389750	13.8388737	4.6762098
H	6.0094107	12.3139652	4.5910409
O	7.6308000	14.4938000	7.1120000
H	7.8097000	14.4568878	8.0707320
H	7.0851850	15.2948805	6.9890226
O	7.7464000	11.5262000	7.0231000
H	8.4521109	11.5911544	6.3550641
H	7.2294822	10.7291512	6.7803227
Ni	-3.5967000	17.6776000	6.7945000
O	-3.4743000	17.4990000	8.8913000
H	-2.5100960	17.6362039	8.9838078
H	-3.5554016	16.5216383	8.8233945
O	-3.8181000	17.8697000	4.7828000
H	-4.1840830	16.9633463	4.6729118
H	-2.8624829	17.7276231	4.5989612
O	-5.5574000	18.0850000	7.1120000
H	-5.6116055	18.3135858	8.0589856
H	-5.9808032	17.2075460	7.0388345
O	-3.0452000	19.6690000	7.0231000
H	-3.3468758	20.2104384	6.2714284
H	-2.0924240	19.5038413	6.8616379
C	6.3001984	8.6027649	5.8880262
H	6.3038153	7.9124628	6.7398881
H	7.3385497	8.8553832	5.6280121
H	5.8421024	8.1099964	5.0222827
H	5.7067015	9.4983966	6.1330328

MOF “B” + H2O

Energy = -6661.665779433

0 1

Ni	-2.7009000	6.7698000	6.7945000
C	0.1049000	11.0412000	6.5634000
C	-1.1923000	11.6628000	6.5481000
C	0.2086000	9.6894000	6.5761000
H	1.1776707	9.1891807	6.5968614
C	-0.9145000	8.8412000	6.5735000
C	-2.2247000	9.4741000	6.5557000

O	-3.2365000	8.6877000	6.5964000
C	-2.2982000	10.8559000	6.5469000
H	-3.3045396	11.2772445	6.5448339
O	-0.8670000	7.5643000	6.6180000
O	-2.6074000	6.9651000	8.8913000
H	-3.2163166	7.7240722	8.9906698
H	-1.7246644	7.3928232	8.8289920
O	-2.7565000	6.4821000	4.7828000
H	-1.7892608	6.6139867	4.6660299
H	-3.1099186	7.3810205	4.5972183
O	-2.0734000	4.8681000	7.1120000
H	-2.2397608	4.7074257	8.0598700
H	-1.1021701	4.9391569	7.0342476
O	-4.7013000	6.2518000	7.0231000
H	-5.0211941	5.7190410	6.2727835
H	-5.0398004	7.1580243	6.8643953
C	1.1956000	13.2937000	6.5634000
C	1.3058000	11.8594000	6.5481000
C	2.3144000	14.0595000	6.5761000
H	2.2623236	15.1483580	6.5975960
C	3.6105000	13.5109000	6.5735000
C	3.7176000	12.0598000	6.5557000
O	4.9045000	11.5767000	6.5964000
C	2.5576000	11.3052000	6.5469000
H	2.6941501	10.2214815	6.5511978
O	4.6926000	14.1905000	6.6180000
C	-1.3004000	13.1120000	6.5634000
C	-0.1135000	13.9246000	6.5481000
C	-2.5231000	13.6981000	6.5761000
H	-3.4401139	13.1081675	6.5967846
C	-2.6960000	15.0949000	6.5735000
C	-1.4929000	15.9131000	6.5557000
O	-1.6680000	17.1825000	6.5964000
C	-0.2594000	15.2858000	6.5469000
H	0.6080166	15.9479409	6.5431812
O	-3.8257000	15.6921000	6.6180000
Ni	6.2976000	12.9995000	6.7945000
O	6.0817000	12.9829000	8.8913000
H	5.5374609	12.1697551	8.9472091
H	5.3968782	13.6871472	8.8297596
O	6.5746000	13.0952000	4.7828000
H	5.7197831	13.5175290	4.5635714
H	6.4943665	12.1476079	4.4911630
O	7.6308000	14.4938000	7.1120000
H	7.8916324	14.5248265	8.0498829
H	7.1234828	15.3111951	6.9467655
O	7.7464000	11.5262000	7.0231000
H	7.4751823	10.8976643	6.2939244
H	7.6122886	11.0265138	7.8457962
Ni	-3.5967000	17.6776000	6.7945000
O	-3.4743000	17.4990000	8.8913000

H	-2.5101648	17.6319923	8.9900223
H	-3.5593070	16.5218580	8.8215857
O	-3.8181000	17.8697000	4.7828000
H	-4.1820791	16.9621258	4.6743578
H	-2.8629045	17.7308312	4.5947538
O	-5.5574000	18.0850000	7.1120000
H	-5.6143448	18.3173160	8.0578709
H	-5.9838180	17.2089614	7.0398050
O	-3.0452000	19.6690000	7.0231000
H	-3.3427641	20.2097610	6.2693272
H	-2.0913189	19.5044798	6.8675843
O	6.3712215	10.4144362	4.9980361
H	6.1459764	9.5850440	4.5504846
H	5.5690681	10.7193523	5.6129605

MOF “B”

Energy = -6585.162369620

Ni	-2.7009000	6.7698000	6.7945000
C	0.1049000	11.0412000	6.5634000
C	-1.1923000	11.6628000	6.5481000
C	0.2086000	9.6894000	6.5761000
H	1.1780411	9.1901982	6.5984585
C	-0.9145000	8.8412000	6.5735000
C	-2.2247000	9.4741000	6.5557000
O	-3.2365000	8.6877000	6.5964000
C	-2.2982000	10.8559000	6.5469000
H	-3.3046862	11.2772230	6.5468884
O	-0.8670000	7.5643000	6.6180000
O	-2.6074000	6.9651000	8.8913000
H	-3.2056053	7.7333049	8.9859087
H	-1.7190270	7.3809608	8.8237554
O	-2.7565000	6.4821000	4.7828000
H	-1.7886672	6.6207989	4.6746781
H	-3.1139562	7.3782732	4.5921851
O	-2.0734000	4.8681000	7.1120000
H	-2.2420608	4.7037659	8.0587860
H	-1.1022217	4.9443075	7.0371460
O	-4.7013000	6.2518000	7.0231000
H	-5.0197532	5.7255321	6.2675373
H	-5.0367083	7.1601474	6.8695233
C	1.1956000	13.2937000	6.5634000
C	1.3058000	11.8594000	6.5481000
C	2.3144000	14.0595000	6.5761000
H	2.2606979	15.1485076	6.5985119
C	3.6105000	13.5109000	6.5735000
C	3.7176000	12.0598000	6.5557000
O	4.9045000	11.5767000	6.5964000
C	2.5576000	11.3052000	6.5469000
H	2.6988207	10.2228687	6.5468894

```
O     4.6926000   14.1905000    6.6180000
C    -1.3004000   13.1120000    6.5634000
C    -0.1135000   13.9246000    6.5481000
C    -2.5231000   13.6981000    6.5761000
H    -3.4400300   13.1080394    6.5989388
C    -2.6960000   15.0949000    6.5735000
C    -1.4929000   15.9131000    6.5557000
O    -1.6680000   17.1825000    6.5964000
C    -0.2594000   15.2858000    6.5469000
H     0.6080194   15.9478852    6.5471027
O    -3.8257000   15.6921000    6.6180000
Ni    6.2976000   12.9995000    6.7945000
O     6.0817000   12.9829000    8.8913000
H     5.7137273   12.0817957    8.9886166
H     5.2786847   13.5458817    8.8227357
O     6.5746000   13.0952000    4.7828000
H     5.9733748   13.8656260    4.6720333
H     5.9737838   12.3388608    4.5971772
O     7.6308000   14.4938000    7.1120000
H     7.8662685   14.4274807    8.0565357
H     7.0773089   15.2960930    7.0441346
O     7.7464000   11.5262000    7.0231000
H     8.3606644   11.5118331    6.2669708
H     7.1246054   10.7840967    6.8691593
Ni   -3.5967000   17.6776000    6.7945000
O    -3.4743000   17.4990000    8.8913000
H    -2.5096612   17.6305654    8.9870813
H    -3.5603878   16.5221430    8.8209407
O    -3.8181000   17.8697000    4.7828000
H    -4.1843179   16.9635247    4.6721734
H    -2.8626298   17.7280612    4.5977152
O    -5.5574000   18.0850000    7.1120000
H    -5.6133012   18.3217498    8.0568421
H    -5.9807260   17.2070014    7.0454248
O    -3.0452000   19.6690000    7.0231000
H    -3.3387000   20.2073092    6.2658960
H    -2.0920210   19.4981515    6.8691545
```

MOF "A" + CH4

Energy = -7311.990241938

```
0 1
Ni   -5.5923943    9.4296309    9.5734817
C    -2.8836288   12.5237736    9.8450015
H    -3.4459890   13.4549936    9.8337583
C    -3.6427344   11.3397521    9.8275606
C    -2.9369038   10.0536125    9.8416670
O    -3.6483558    8.9724498    9.8538812
O    -4.9367172   11.3199305    9.8226261
```

O	-5.3104472	9.5361248	7.4383822
H	-6.1555951	9.9512919	7.1800611
H	-5.4085027	8.5891704	7.2209870
C	-1.3829480	14.9966779	9.8770134
H	-2.4687249	15.0663847	9.8812434
C	0.7829095	16.1835098	9.8572810
C	-0.6832348	16.2166592	9.8645425
O	-1.2624948	17.3740756	9.8793541
O	1.4137202	17.3140709	9.8645806
O	-5.8692401	9.3143833	11.7080723
H	-5.7646617	10.2602803	11.9271023
H	-5.0213551	8.8964600	11.9532688
C	-10.3635439	7.6311440	9.0700626
C	-9.6826275	6.3806482	9.0930495
C	-9.6706683	8.8228802	9.1329217
H	-10.1802897	9.7869928	9.1185569
C	-8.3066239	6.3181138	9.1788508
H	-7.7734748	5.3668771	9.1992385
C	-8.2523989	8.8119406	9.2274127
C	-7.5473733	7.5178435	9.2509674
O	-6.2570456	7.5382194	9.3163101
O	-7.5365380	9.8867415	9.2738799
C	0.8913896	23.8596814	9.0619716
C	-0.5323452	23.8818348	9.0764363
C	1.5878924	22.6703573	9.1315369
H	2.6779168	22.6410924	9.1241609
C	-1.2639739	22.7147096	9.1596319
H	-2.3544479	22.7180338	9.1739152
C	0.8801291	21.4416083	9.2242296
C	-0.5929548	21.4639120	9.2384698
Ni	5.3919106	9.1948597	9.5711417
C	0.6697552	11.2108117	9.8605110
C	1.4226325	12.4514320	9.8621238
C	1.3599607	9.9963918	9.8497264
H	0.8346669	9.0436732	9.8426944
C	2.8185969	12.4005608	9.8558723
H	3.4204332	13.3066408	9.8555100
C	2.7645209	9.9307100	9.8304877
C	3.5258383	11.1849165	9.8353918
O	4.8177205	11.1094612	9.8422919
O	3.4280875	8.8194735	9.8306139
O	5.1404269	9.3673252	7.4373288
H	5.1953745	8.4246273	7.1892404
H	6.0068416	9.7503172	7.2005217
C	0.7207794	13.7299535	9.8672625
C	-0.7299228	13.7616782	9.8693106
C	1.4279241	14.9346204	9.8667026
H	2.5155585	14.9546922	9.8608606
C	-1.4867178	12.5146065	9.8603041
C	-0.7884773	11.2423694	9.8631748
C	-1.5306662	10.0587505	9.8630443

H	-1.0465601	9.0845598	9.8677750
O	5.6346313	9.0403282	11.7073992
H	4.7621420	8.6645487	11.9339710
H	5.5735174	9.9890101	11.9313327
C	9.3249229	5.9494251	9.0760383
C	10.0702461	7.1628313	9.0893069
C	7.9466251	5.9573051	9.1434626
H	7.3641666	5.0352393	9.1362121
C	9.4397701	8.3878626	9.1691171
H	9.9996032	9.3236354	9.1817979
C	7.2504333	7.1935312	9.2329297
C	8.0216194	8.4494749	9.2449210
O	7.3614611	9.5589637	9.3042740
O	5.9622969	7.2795444	9.2843066
Ni	0.1054883	18.8249574	9.5888423
O	0.1357338	19.1503441	11.7185999
H	0.9057764	18.5937845	11.9449090
H	-0.6468811	18.6216858	11.9672684
O	0.0920875	18.4856579	7.4598155
H	0.8770435	19.0078496	7.2055323
H	-0.6769993	19.0316529	7.2074852
O	-1.2098401	20.3305202	9.3042032
O	1.4629539	20.2891183	9.2783133
H	1.4358358	24.8027275	8.9959175
H	-1.0472843	24.8420625	9.0222895
H	-11.4523117	7.6405419	9.0034187
H	-10.2647856	5.4594360	9.0435975
H	11.1588851	7.1162490	9.0370992
H	9.8590489	5.0002057	9.0132033
C	4.6502001	18.7349992	9.7351524
H	4.0072258	17.8479086	9.7493636
H	5.2342760	18.7863874	10.6624068
H	5.3351663	18.6841825	8.8797306
H	4.0115420	19.6211902	9.6472130

MOF “A” + CO2

Energy = -7460.154183311

0 1

Ni	-5.5720844	9.4519937	9.5233477
C	-2.8396270	12.5345789	9.7213838
H	-3.3955575	13.4694159	9.6979023
C	-3.6071198	11.3555873	9.7230267
C	-2.9103275	10.0653617	9.7699195
O	-3.6293800	8.9895764	9.7999789
O	-4.9011836	11.3452229	9.7138883
O	-5.3218780	9.5129816	7.3832788
H	-6.1675622	9.9306410	7.1306685
H	-5.4290958	8.5640213	7.1801497
C	-1.3208396	14.9967713	9.6825832

H	-2.4029497	15.0706648	9.5962614
C	0.8476856	16.1712872	9.8307279
C	-0.6132237	16.2106783	9.7094810
O	-1.1796462	17.3729672	9.6571285
O	1.4833762	17.2964298	9.8885107
O	-5.8230247	9.4014651	11.6642000
H	-5.7198756	10.3543202	11.8514947
H	-4.9701691	8.9951386	11.9115965
C	-10.3536623	7.6488598	9.1497696
C	-9.6731953	6.3985155	9.1900146
C	-9.6591122	8.8412167	9.1712317
H	-10.1688818	9.8049783	9.1428774
C	-8.2959288	6.3368737	9.2521249
H	-7.7628811	5.3858977	9.2854015
C	-8.2392516	8.8314682	9.2393554
C	-7.5349930	7.5373641	9.2819159
O	-6.2437793	7.5577091	9.3239524
O	-7.5215722	9.9061478	9.2452421
C	0.9629041	23.8684313	9.0952241
C	-0.4608127	23.8765184	9.0765854
C	1.6689602	22.6851371	9.1591121
H	2.7590680	22.6650427	9.1788361
C	-1.1824363	22.7005620	9.1217914
H	-2.2729024	22.6950018	9.1060045
C	0.9719741	21.4465117	9.2167822
C	-0.5014634	21.4569116	9.1926882
Ni	5.4176877	9.1580089	9.5790173
C	0.7037873	11.1985659	9.8101786
C	1.4641737	12.4342774	9.8392828
C	1.3863073	9.9798131	9.7971733
H	0.8550209	9.0310186	9.7620390
C	2.8596331	12.3745557	9.8568987
H	3.4668816	13.2770019	9.8660467
C	2.7907880	9.9054539	9.8062123
C	3.5593491	11.1549560	9.8373046
O	4.8505696	11.0710176	9.8623022
O	3.4477292	8.7902592	9.8070439
O	5.1901123	9.3357810	7.4424035
H	5.2389588	8.3913224	7.1995040
H	6.0636706	9.7083772	7.2154921
C	0.7702625	13.7172236	9.8319142
C	-0.6777926	13.7580093	9.7501918
C	1.4827292	14.9172479	9.8877896
H	2.5676506	14.9313561	9.9666062
C	-1.4430164	12.5162288	9.7488808
C	-0.7540240	11.2396593	9.7897751
C	-1.5044753	10.0614760	9.8074287
H	-1.0271177	9.0847429	9.8464817
O	5.6367427	8.9795278	11.7153761
H	4.7651072	8.5939584	11.9282288
H	5.5667594	9.9243189	11.9525863

C	9.3536825	5.9121774	9.1115869
C	10.0994691	7.1251122	9.1352966
C	7.9747538	5.9205815	9.1653387
H	7.3920425	4.9988155	9.1503542
C	9.4688198	8.3501713	9.2128656
H	10.0287563	9.2857258	9.2344521
C	7.2781814	7.1567497	9.2508036
C	8.0500100	8.4120674	9.2752820
O	7.3898522	9.5215569	9.3328285
O	5.9895263	7.2432398	9.2882435
Ni	0.2191251	18.8168001	9.5344042
O	0.0696806	19.2116185	11.6260716
H	0.5210127	18.4534581	12.0395135
H	-0.8893975	19.0900010	11.7950974
O	0.3425010	18.5046751	7.3991513
H	1.0995002	19.0894603	7.2018037
H	-0.4487416	18.9926656	7.1018171
O	-1.1084987	20.3143503	9.2172764
O	1.5670653	20.3020381	9.2712599
H	1.4987926	24.8179715	9.0608434
H	-0.9852330	24.8317957	9.0265436
H	-11.4434896	7.6578246	9.1029592
H	-10.2565846	5.4768620	9.1732311
H	11.1885577	7.0782342	9.0934711
H	9.8877739	4.9627669	9.0519152
C	-3.3445663	18.9669218	10.4144383
O	-2.8470077	19.0875276	11.4735143
O	-3.9027119	18.8565169	9.3969937

MOF “A” + CO2 (Local Minimum)

Energy = -7460.144399288

Ni	-5.5907725	9.4442297	9.5653852
C	-2.8729434	12.5259944	9.8429491
H	-3.4323835	13.4590619	9.8422011
C	-3.6363950	11.3452465	9.8201641
C	-2.9356022	10.0563563	9.8332584
O	-3.6505309	8.9776300	9.8411182
O	-4.9304696	11.3316942	9.8121453
O	-5.3184484	9.5514860	7.4283023
H	-6.1681247	9.9620854	7.1773512
H	-5.4115740	8.6049175	7.2076100
C	-1.3623188	14.9948000	9.8772081
H	-2.4480232	15.0677530	9.8883895
C	0.8103959	16.1705935	9.8641846
C	-0.6562545	16.2120918	9.8756144
O	-1.2272374	17.3734402	9.9085303
O	1.4499827	17.2958274	9.8892028
O	-5.8584289	9.3460123	11.7036664
H	-5.7443660	10.2937344	11.9103194

H	-5.0131783	8.9238299	11.9505100
C	-10.3609731	7.6466307	9.0823377
C	-9.6794715	6.3964752	9.1075316
C	-9.6688657	8.8390948	9.1415300
H	-10.1792169	9.8028597	9.1281192
C	-8.3035592	6.3348792	9.1917650
H	-7.7690234	5.3844753	9.2164053
C	-8.2503895	8.8291112	9.2309421
C	-7.5451245	7.5351526	9.2568881
O	-6.2546726	7.5551728	9.3177862
O	-7.5346068	9.9042880	9.2704606
C	1.0046517	23.8403206	9.0701684
C	-0.4189084	23.8758530	9.0730513
C	1.6902358	22.6454897	9.1553227
H	2.7802612	22.6077500	9.1548954
C	-1.1610018	22.7156786	9.1570268
H	-2.2514409	22.7267062	9.1605191
C	0.9710077	21.4224449	9.2519246
C	-0.5021907	21.4601487	9.2499798
Ni	5.3995136	9.1748034	9.5840246
C	0.6755160	11.1975733	9.8501301
C	1.4317988	12.4357856	9.8451780
C	1.3622161	9.9804332	9.8512827
H	0.8328769	9.0297518	9.8554221
C	2.8271129	12.3798000	9.8418038
H	3.4317844	13.2833529	9.8392305
C	2.7673896	9.9101263	9.8374926
C	3.5310331	11.1629577	9.8326399
O	4.8229932	11.0863894	9.8484231
O	3.4307412	8.7986366	9.8538580
O	5.1481120	9.3353642	7.4505319
H	5.1912455	8.3882343	7.2171372
H	6.0184943	9.7037985	7.2054668
C	0.7350834	13.7169530	9.8514583
C	-0.7150747	13.7562390	9.8587596
C	1.4476276	14.9179270	9.8572850
H	2.5350682	14.9324571	9.8484754
C	-1.4761781	12.5117066	9.8512197
C	-0.7826380	11.2362735	9.8539949
C	-1.5296023	10.0558985	9.8595390
H	-1.0496155	9.0798048	9.8728240
O	5.6547190	8.9988008	11.7125765
H	4.7821490	8.6206210	11.9353068
H	5.5964414	9.9438308	11.9510255
C	9.3356664	5.9333886	9.0888707
C	10.0811145	7.1467239	9.0996565
C	7.9575827	5.9420319	9.1563250
H	7.3736031	5.0209778	9.1484719
C	9.4512387	8.3721095	9.1803661
H	10.0123548	9.3072413	9.1894425
C	7.2618896	7.1781692	9.2453753

C	8.0327980	8.4343484	9.2591874
O	7.3718652	9.5431786	9.3205047
O	5.9734577	7.2624981	9.2946173
Ni	0.1587240	18.8165937	9.6156646
O	0.1665335	19.1588571	11.7385660
H	0.9278866	18.5958297	11.9770963
H	-0.6249371	18.6378997	11.9756663
O	0.1443105	18.4814391	7.4864184
H	0.9211123	19.0143924	7.2295105
H	-0.6327247	19.0205810	7.2439071
O	-1.1334144	20.3343667	9.3150869
O	1.5411087	20.2646608	9.3206284
H	1.5573749	24.7783783	8.9997591
H	-0.9259265	24.8397477	9.0090286
H	-11.4497878	7.6552914	9.0173156
H	-10.2610507	5.4747268	9.0617479
H	11.1696229	7.0997024	9.0434749
H	9.8693002	4.9837539	9.0272589
O	5.5642831	15.5644640	9.7238818
C	5.4877113	15.5667689	10.8923156
O	5.4098706	15.5698351	12.0611611

MOF "A" + N2

Energy = -7381.035568786

Ni	-5.5925889	9.4409652	9.5767021
C	-2.8725740	12.5247225	9.8479556
H	-3.4310457	13.4582671	9.8379952
C	-3.6363666	11.3438517	9.8310933
C	-2.9355250	10.0549717	9.8444896
O	-3.6511960	8.9767295	9.8571608
O	-4.9304991	11.3293028	9.8273466
O	-5.3073541	9.5532339	7.4418645
H	-6.1526293	9.9685881	7.1842137
H	-5.4026852	8.6077075	7.2176181
C	-1.3616544	14.9924210	9.8789439
H	-2.4471815	15.0667023	9.8860675
C	0.8099512	16.1697610	9.8550168
C	-0.6564480	16.2093448	9.8667082
O	-1.2308423	17.3691755	9.8861962
O	1.4475924	17.2962371	9.8638318
O	-5.8755547	9.3347699	11.7112496
H	-5.7672222	10.2818435	11.9236301
H	-5.0313473	8.9144428	11.9644939
C	-10.3638285	7.6454223	9.0631620
C	-9.6832877	6.3947090	9.0874263
C	-9.6708944	8.8371465	9.1285312
H	-10.1806721	9.8012222	9.1137164
C	-8.3076011	6.3319595	9.1775114
H	-7.7744827	5.3807265	9.1996965

C	-8.2527702	8.8260360	9.2261049
C	-7.5483592	7.5315975	9.2517185
O	-6.2582999	7.5512785	9.3206891
O	-7.5363372	9.9004879	9.2737806
C	0.9962057	23.8341666	9.0654410
C	-0.4271544	23.8741123	9.0740899
C	1.6774867	22.6364276	9.1403481
H	2.7671226	22.5929084	9.1372146
C	-1.1735085	22.7163204	9.1567197
H	-2.2638721	22.7335759	9.1669836
C	0.9543750	21.4162391	9.2331321
C	-0.5186345	21.4575751	9.2409750
Ni	5.3927347	9.1688808	9.5713928
C	0.6757656	11.1969500	9.8591807
C	1.4324769	12.4349186	9.8555270
C	1.3624089	9.9801765	9.8520124
H	0.8343223	9.0289974	9.8493171
C	2.8279374	12.3793792	9.8456166
H	3.4317844	13.2833529	9.8392305
C	2.7669761	9.9102138	9.8312280
C	3.5316684	11.1623120	9.8297060
O	4.8234483	11.0841511	9.8358762
O	3.4278196	8.7971811	9.8349361
O	5.1404370	9.3348637	7.4365306
H	5.2095944	8.3917407	7.1934053
H	6.0012634	9.7293110	7.1983401
C	0.7357461	13.7159787	9.8613736
C	-0.7144176	13.7542627	9.8680498
C	1.4480210	14.9172172	9.8590732
H	2.5350682	14.9324571	9.8484754
C	-1.4757402	12.5100602	9.8607569
C	-0.7823300	11.2348845	9.8634152
C	-1.5292627	10.0544388	9.8648770
H	-1.0489489	9.0783308	9.8696353
O	5.6346011	9.0074626	11.7069408
H	4.7601863	8.6341068	11.9304912
H	5.5787529	9.9541693	11.9396101
C	9.3337372	5.9340307	9.0693411
C	10.0756970	7.1494065	9.0831840
C	7.9554704	5.9379032	9.1383938
H	7.3757539	5.0141824	9.1306064
C	9.4418990	8.3724333	9.1657981
H	9.9989677	9.3098207	9.1790352
C	7.2558585	7.1719649	9.2303854
C	8.0237708	8.4298663	9.2437463
O	7.3609764	9.5375176	9.3060823
O	5.9674778	7.2542873	9.2829534
Ni	0.1465445	18.8120836	9.5951743
O	0.1706211	19.1311901	11.7267872
H	0.9365049	18.5699470	11.9553642
H	-0.6159110	18.6041852	11.9666996

O	0.1239512	18.4784074	7.4638247
H	0.9183573	18.9866897	7.2109204
H	-0.6346571	19.0423833	7.2191159
O	-1.1507767	20.3323860	9.3065065
O	1.5231240	20.2573158	9.2927500
H	1.5523451	24.7703544	9.0005402
H	-0.9305816	24.8402115	9.0163398
H	-11.4524534	7.6550488	8.9936400
H	-10.2653880	5.4735228	9.0357020
H	11.1643175	7.1061381	9.0292116
H	9.8703861	4.9864497	9.0048145
N	5.5669841	15.5643568	9.6822690
N	5.4955747	15.5671920	10.7824508
